# Antagonistic Functions of Androgen Receptor and NF-κB in Prostate Cancer—Experimental and Computational Analyses

**DOI:** 10.3390/cancers14246164

**Published:** 2022-12-14

**Authors:** José Basílio, Bernhard Hochreiter, Bastian Hoesel, Emira Sheshori, Marion Mussbacher, Rudolf Hanel, Johannes A. Schmid

**Affiliations:** 1 Center for Physiology and Pharmacology, Institute of Vascular Biology and Thrombosis Research, Medical University Vienna, Schwarzspanierstraße 17, 1090 Vienna, Austria; 2 INESC ID—Instituto de Engenharia de Sistemas e Computadores, Investigação e Desenvolvimento em Lisboa, Universidade de Lisboa, Rua Alves Redol 9, 1000-029 Lisboa, Portugal; 3 Department of Pharmacology and Toxicology, University of Graz, 8010 Graz, Austria; 4 Complexity Science Hub Vienna, Josefstaedter Strasse 39, 1080 Vienna, Austria; 5 Section for Science of Complex Systems, Medical University of Vienna, Spitalgasse 23, 1090 Vienna, Austria

**Keywords:** prostate cancer, androgen receptor, NF-κB, PTEN, fluorescence microscopy, gene expression, gene set enrichment analysis, network analysis

## Abstract

**Simple Summary:**

The standard treatment of metastasizing prostate cancer by androgen receptor blockers fails to provide a curative therapy, with most of the patients dying from tumor relapse. We show by experimental and computational methods that androgen receptor blockade upregulates inflammatory pathways, which may finally trigger survival mechanisms of cancer cells. Therefore, we hypothesize that a combined anti-inflammatory/antiandrogen treatment might be beneficial and should be tested in preclinical models as well as clinical studies of prostate cancer.

**Abstract:**

Prostate cancer is very frequent and is, in many countries, the third-leading cause of cancer related death in men. While early diagnosis and treatment by surgical removal is often curative, metastasizing prostate cancer has a very bad prognosis. Based on the androgen-dependence of prostate epithelial cells, the standard treatment is blockade of the androgen receptor (AR). However, nearly all patients suffer from a tumor relapse as the metastasizing cells become AR-independent. In our study we show a counter-regulatory link between AR and NF-κB both in human cells and in mouse models of prostate cancer, implying that inhibition of AR signaling results in induction of NF-κB-dependent inflammatory pathways, which may even foster the survival of metastasizing cells. This could be shown by reporter gene assays, DNA-binding measurements, and immune-fluorescence microscopy, and furthermore by a whole set of computational methods using a variety of datasets. Interestingly, loss of PTEN, a frequent genetic alteration in prostate cancer, also causes an upregulation of NF-κB and inflammatory activity. Finally, we present a mathematical model of a dynamic network between AR, NF-κB/IκB, PI3K/PTEN, and the oncogene c-Myc, which indicates that AR blockade may upregulate c-Myc together with NF-κB, and that combined anti-AR/anti-NF-κB and anti-PI3K treatment might be beneficial.

## 1. Introduction

Prostate cancer is one of the most frequent malignancies, and about 1.4 million cases are reported each year [1]. When diagnosed at an early stage, treatment strategies vary between active surveillance (or “watchful waiting”), radiation therapy, and surgical removal of the whole prostate [2]. The latter is the preferred treatment option in Europe and is generally considered curative as long as the cancer remained organ-confined. However, metastasizing prostate cancer still poses a major problem as it is very hard to cure, leading to an estimated number of 375,000 deaths worldwide in the year 2020 [1]. Treatment with androgen receptor antagonists or interfering with the synthesis of androgens remains the standard therapy for patients with advanced or metastatic forms of prostate cancer, as the prostate epithelium regresses after removal of androgens or blockade of the androgen receptor (AR) [3,4,5]. Accordingly, androgen deprivation therapy (ADT) leads to a regression of the cancer and an alleviation of the tumor burden in most cases. However, a major drawback of this therapy is that virtually all patients develop an androgen-independent, “castration-resistant prostate cancer (CRPC)” after some time, which is characterized by a relapse of tumor growth and new metastatic spread, and in most cases eventually leads to the patient’s death [6]. The initial cancer regression is usually monitored by a reduction of the disease biomarker PSA (prostate-specific antigen) in the blood. However, it must be stated that a decrease in PSA levels does not necessarily mean a reduction of cancer cells. It just shows that AR signaling is reduced, as PSA is an androgen-dependent gene. In fact, the androgen dependence of prostate epithelial cells and cancer cells derived from them is more complex. While androgen deprivation was reported to result at least in a transient reduction of cell proliferation [4], other reports claimed that AR is driving differentiation of normal prostate epithelial cells rather than proliferation [7]. Furthermore, it could be observed that androgens have different effects in vitro as compared to the situation in vivo. In vitro, AR signaling actually suppressed the proliferation of human prostate epithelial progenitor cells in parallel with a reduction of c-Myc levels. On the other hand, a tissue recombination model of stromal cells and human prostate epithelial progenitor cells implanted into the renal capsule implied a positive effect of AR overexpression on epithelial cell proliferation in vivo [8]. Thus, the effect of androgen deprivation apparently depends substantially on stroma/epithelium crosstalk, and it has been postulated that stromal and epithelial activation of AR maintains the differentiated state of the cells and inhibits organ growth in a healthy individual [8]. Furthermore, there seem to be different pathways for how prostate cancer cells can evade androgen deprivation. One important path is via amplification of AR so that it responds even to low levels of androgens (e.g., produced by the tumor itself); another route is via mutations of AR in a way that it becomes persistently active even in the absence of androgens. This might include mutations, which lead to an activation of AR by the antiandrogen drugs themselves. A further possibility is that AR signaling is bypassed or overruled by other pathways which drive proliferation or inhibit apoptosis of prostate cancer cells [6,9,10]. Indeed, accumulating evidence suggests that, besides AR signaling, other pathways play a role for the development of androgen-independent cancer progression [9]. One of the most interesting pathways in this context is the NF-κB signaling pathway.

**Figure 1:**
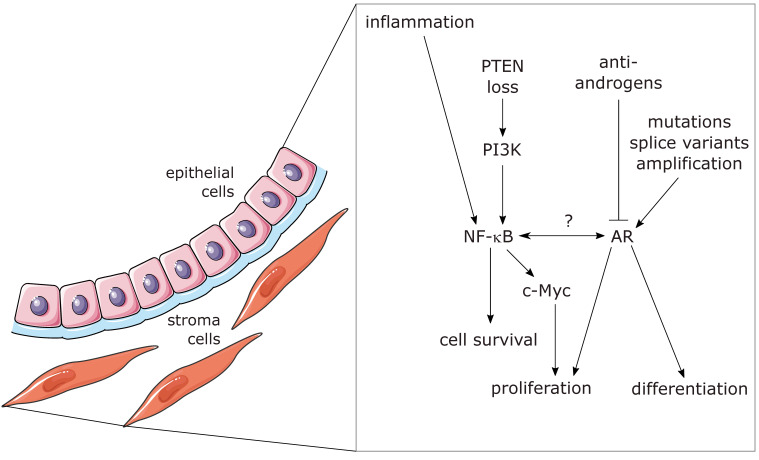
Schematic illustration of links between AR, NF-κB, other relevant signaling molecules, and biological processes in prostate glands.

The term NF-κB, abbreviated from “nuclear factor regulating the kappa-light chain of immunoglobulins in B-cells”, designates a family of transcription factors which is in nonactivated cells usually not localized in the nucleus, but in the cytosol, where it is bound to inhibitory proteins of the IκB family (inhibitors of NF-κB). Furthermore, it not only regulates the kappa-light chain in B-cells, but a great variety of target genes in basically all human cells. NF-κB is not a single protein, but a dimer formed by two of the five NF-κB family members (RelA/p65, RelB, c-Rel, NFKB1/p105, and NFKB2/p100). All these five proteins contain a common homology domain, which drives dimerization of the family members and furthermore binds specific DNA sequences in promoter/enhancer regions of NF-κB target genes [11,12]. Three of the NF-κB members (RelA, RelB, and c-Rel) contain a transactivation domain, while the other two (p105 and p100) lack such a region, but contain an inhibitory domain, which has to be phosphorylated and proteolytically cleaved to give rise to the mature protein forms p50 and p52, respectively. Homo- and heterodimers of p50 and p52 bind to DNA but cannot induce transcription and therefore act as repressors. However, dimers containing at least one member with a transactivation domain are able to recruit the transcriptional machinery and thus usually induce expression of downstream target genes. NF-κB transcription factors represent the central signaling hubs for inflammatory processes. They are present in all human cells, but have also specific roles in immune cell development and regulation [13]. In general, inflammation is a physiological response to cope with all different kinds of stress, which can be triggered by pathogens, but also by other “danger signals” or physical strains such as irradiation or heat. A whole variety of signaling pathways originating either from cell surface receptors or from intracellular sensors is able to activate NF-κB. Most of these pathways converge at an enzyme complex, termed IKK (IκB kinase)-signalosome, which upon activation phosphorylates inhibitory IκB molecules on two adjacent serine residues, triggering them for K48-linked polyubiquitination and subsequent degradation by proteasomes [14]. This releases NF-κB dimers from their inhibitors, leading to nuclear translocation and induction of target genes. Depending on the cell type and epigenetic state, different genes can be induced at different levels. Some of these genes are inflammatory cytokines and chemokines creating a feedforward loop, while others, such as adhesion factors, are important for leukocyte recruitment during the immune response against the endangering stressor. Moreover, antiapoptotic genes are often upregulated to provide cellular survival mechanisms—so that the cells have the possibility to cope with the stress, until defense and repair mechanism restore the original state. Importantly, NF-κB also induces its own inhibitors of the IκB family, generating a negative feedback loop, whose task is to finally inactivate the inflammatory signaling and to switch the cells back to the nonactivated state. However, in many cases, this deactivation is not complete, leaving the cells or the affected tissue in a chronic inflammatory state, which can last for longer periods of time. In the case that oncogenic mutations occur within that timeframe, the higher levels of antiapoptotic genes induced by constitutive active NF-κB can contribute to the survival of transformed cells, which would otherwise undergo oncogene-induced senescence or apoptosis [12]. We hypothesized that androgen deprivation, as usually performed for metastasizing prostate cancer, may cause a stress situation for prostate cancer cells, which might upregulate NF-κB activity and thereby cellular survival mechanisms. Indeed, prior observations already indicated a negative effect of AR on NF-κB signaling, as activation of AR was associated with a downregulation of NF-κB activity [15]. At least in part this could be explained by an androgen-mediated maintenance of the NF-κB-inhibitory IκBα protein under conditions where it would be normally degraded, leading to release of active NF-κB [16]. A link between AR and NF-κB signaling is also indicated by the fact that androgen-independent prostate cancer cell lines and xenografts often exhibit an elevated constitutive NF-κB activity [17,18]. Furthermore, an increased rate of apoptosis in AR expressing cells has been shown to be related to decreased NF-κB activity [19]. Contrary to the negative effect of AR on NF-κB activity, it has been reported that NF-κB can increase expression and activity of AR or its splice-variant AR-V7, which led to the concept that NF-κB and AR act in a cooperative manner [20,21]. However, increased cell survival after induction of NF-κB concomitant with a higher expression of AR does not necessarily mean that AR activity contributes to cell viability. Generally, the link between AR and NF-κB seems complex, as other proteins that are important in prostate cancer may also be involved in a crosstalk between these transcription factors. It is known, for example, that the oncogene c-Myc, which is upregulated in a high percentage of prostate cancers, is induced by NF-κB [22]; and that this oncogene is stabilized by IKKα, one of the inflammatory enzymes that play a role in NF-κB activation [23]. Another gene with a crucial role in prostate cancer development is the phosphatase PTEN, which is mutated or lost in up to 30% of the cases. It has been shown that loss of this tumor suppressor increases NF-κB activity in vivo, which can lead to invasive prostate carcinoma when combined with further mutations [24]. For an overview of links between NF-κB, AR, PTEN, and c-Myc, see Figure 1. While there are many reports demonstrating an elevated NF-κB activity in prostate cancer, there were hardly any clinical attempts to inhibit that signaling pathway therapeutically, most likely because NF-κB is also crucial for immune cell functions, which are considered essential for tumor immune surveillance—and which should obviously not be hampered. Our aim was to investigate the AR/NF-κB axis in more detail by using two different human prostate cancer cell lines, as well as a mouse model of prostate cancer, experimentally and to combine that with computational meta-analyses of several publicly available datasets. We focused on the AR-positive cell lines VCaP and LNCaP, because other AR-independent or -negative cells such as DU145 or PC3 are so drastically dedifferentiated that they do not reflect early stages of metastasizing prostate cancer. We confirm that an activation of AR signaling leads to a repression of NF-κB activity and, vice versa, that an AR inhibition results in an upregulation of NF-κB and inflammatory pathways without significant induction of cell death but rather an activation of cellular survival mechanisms. Altogether, this suggests that combined blockade of AR and NF-κB might be beneficial for treatment of metastasizing prostate cancer.

These processes can occur differently in epithelial and stromal cells and can also be influenced by paracrine signaling between the cells involved (smooth-muscle stroma cells and epithelial cell illustration: by Servier, https://smart.servier.com/, licensed under CC-BY 3.0 Unported: https://creativecommons.org/licenses/by/3.0/) (accessed on 7 November 2022).

## 2. Materials and Methods

### 2.1. Cell Culture and Materials

The human prostate carcinoma cell lines LNCAP and VCAP were both obtained from ATCC. Cells were routinely cultured in RPMI and DMEM medium containing 10% fetal calf serum (FBS), respectively. Cells were maintained at 37 °C in a humidified atmosphere of 5% CO_2_. For androgen depletion and restimulation of the AR signaling axis, LNCAPs and VCAPs were seeded at 50–60% density in RPMI medium or DMEM, without phenol red, containing 5% charcoal-stripped FBS (Sigma, Vienna, Austria) to minimize potential androgens. Transient transfections of all reporter and expression plasmids were performed with Turbofect (Thermo Fisher Scientific, Vienna, Austria), according to the manufacturer’s instructions, on the second day of starvation. After 3 days of starvation, cells were stimulated with the indicated concentrations of dihydrotestosterone (DHT, obtained from Sigma, Vienna, Austria) and analyzed at the indicated time points.

### 2.2. ABCD Assay

Avidin–biotin complex DNA (ABCD) assay was performed as previously described [25]. Briefly, cells were lysed in NETN Buffer (10 mM Tris pH 8.0; 100 mM NaCl; 1 mM EDTA pH 8.0; 10% glycerol; 0.5% Nonidet; 1 mM DTT) and incubated for 5 min at 37 °C with Buffer H (20 mM HEPES pH 7.8; 50 mM KCl; 20% glycerol; 0.1% Nonidet; 1 mM DTT) and the respective biotinylated oligonucleotides (+/− nonbiotinylated competitor oligonucleotides as specificity controls) followed by an incubation for 1 h on ice. After addition of streptavidin agarose, the beads were washed several times with Buffer H and the bound protein was isolated using Laemmli Buffer. Detection was performed using Western blotting with anti-p65 antibodies (Santa Cruz, SC-109). Specificity of the biotinylated oligonucleotides was assured by a negative control (using no oligonucleotides) and a competitor control (using a tenfold excess of nonbiotinylated oligonucleotides) in previous experiments. Oligonucleotides used in this study were:

NF-κB_for:

Bio-GGGAAATTCCCTTGGAAATTCCCTTGGAAATTCCCCTTGGAAATTCC,

NF-κB_rev:

Bio-GGAATTTCCAAGGGGAATTTCCAAGGGAATTTCCAAGGGAATTTCCC.

### 2.3. Reporter Gene Assays

Luciferase reporter gene assays were performed according to a protocol previously described [26]. Briefly, cells were transfected one day after seeding in androgen-depleted medium and starvation was carried out for two additional days. After 24 h of DHT treatment, cells were lysed and luciferase activity was measured using a Biotek Synergy H4 reader.

### 2.4. Western Blot

Western Blot was performed using antibodies: p65 (cell sign. # 8242 or sc-109), p105/p50 (cell sign. # 3035), p100/p52 (cell sign. # 4882), RelB (cell sign. # 4992), c-Rel (cell sign. # 4727), IκBα (sc-371), p-p65 (Ser536) (cell sign. # 3033) and ß-Tubulin (sc-9104). Quantification of Western blots was performed using the Analyze-Gels function of ImageJ.

### 2.5. Staining of Mouse Prostate Tissue for AR and p65-NF-κB

FFPE mouse prostate tissue slides were deparaffinized in xylol, followed by a decreasing row of 90%, 80%, 70%, 50%, and 0% ethanol. Antigen retrieval was achieved by 30 min boiling in pH9 Tris-EDTA buffer and tissues were blocked in 1% BSA in TBS buffer solution supplemented with 0.1% Tween. Antibodies used for staining were as follows: p65 (novusbio; Alexa405 labeled monoclonal IgG1k mouse antibody; prod. no. NBP2-27416AF405; dilution 1:500); and AR (Millipore; polyclonal rabbit antibody; prod. no. 06-680; dilution 1:100; followed by: Abcam; Dylight 650 labeled polyclonal donkey anti rabbit IgG antibody; prod. no. ab96922; dilution 1:1000). Tissue sections were stained overnight, followed by a 5 min counterstaining with DAPI. Tissues were imaged on a Nikon A1 laser scanning confocal microscope in full spectral mode. Specific stainings were isolated from the spectral data via a custom linear unmixing routine in Fiji/ImageJ and intensity was measured per cell, supported by a custom automated cell-segmentation routine. The commercial IKOSA-platform of the company KML-Vision (www.kmlvision.com) was used for image handling, cell recognition, and quantification of fluorescence intensities.

### 2.6. Transcriptomics Analysis

Datasets used in this manuscript were GSE8702 [27], GSE56188, GSE62473, GSE5901 [28], GSE24691 [29], and GSE26410 [30]. Microarrays were routinely analyzed using Bioconductor (v3.15 [31] and R (v4.2.0) packages [32]. Datasets GSE62473 and GSE24691 were analyzed as follows: the non-normalized datasets were retrieved from the GEO database using the GEOquery package (v2.64.2 [33]). Afterwards, the expression profiles for regular probes, were read using the *read.ilm* function from limma package (v3.52.2 [34]), which allows that the detection *p*-values as well as the expression values are read. The *neqc* function performs normexp background correction using negative controls, followed by quantile normalization and finally log2 transformation. It also automatically removes the control probes, leaving only the regular probes. Probes that are expressed in at least half of the arrays according to detection *p*-values of 5% were kept. The illuminaHumanv4.db (v1.26.0 [35]) package and the GPL6885 full table were used to annotate those Illumina ProbeIDs to EntrezGeneIDs and Gene Symbol of datasets GSE62473 and GSE24691, respectively. The Illumina probes were collapsed to EntrezGeneIDs by selecting the probe with the maximum mean intensity value for each probe.

Datasets GSE8702, GSE56188, GSE5901, and GSE26410 were analyzed as follows: the raw CEL files were retrieved from the ArrayExpress database using the ArrayExpress package (v1.56.0 [36]). The CEL files were read using the *read.affy* function from the affy package (v1.74.0 [37]), and the data were quality controlled with the arrayQualityMetrics package (v.3.52.0 [38]). Furthermore, the expression dataset was normalized using the *rma* function from the affy package (v1.74.0 [37]). The probes were annotated with an in-house script, and those probes without EntrezGeneIDs were removed using the *featureFilter* function of the genefilter package (v1.78.0 [39]).

### 2.7. Ingenuity Pathway Analysis (IPA)

Differentially expressed genes (adjusted *p*-value < 0.05) were uploaded into Ingenuity Pathway Analysis software to further obtain information on the biological context of the datasets studied [40] (IPA, QIAGEN Inc.). For IPA analysis, *p*-values were calculated using Fisher’s exact test. The analysis included only the functions and pathways with *p*-value < 0.05.

### 2.8. Gene Set Enrichment Analysis and Overrepresentation Analysis

Gene set enrichment analysis (GSEA) [41] was used to interpret biologically the studied datasets. We used both the software package from the Broad Institute [41] using the Hallmark gene sets [42] and R-scripts for GSEA as described here. Principally, GSEA needs a list of ranked genes. Since *p*-value and log-fold changes (logFC) are often used to evaluate significant results from differential expression analysis, and the up/downregulated genes are usually at the top/bottom of the ranked gene list, we use the signed log10 (*p*-value) to rank genes, where the sign is from logFC [43]. Overrepresentation analysis (ORA) [44] is a widely used approach to determine whether known biological functions are overrepresented (=enriched) in an experimentally-derived gene list, e.g., a list of differentially expressed genes. The analysis was performed with the genes that show an adjusted *p*-value < 0.05 and an absolute log2FC > 1. Both GSEA and ORA analysis were performed using the clusterProfiler package of R Bioconductor (v4.4.4 [45]). The list of AR-induced genes and AR-repressed genes was previously published [46]. The list of NF-κB signaling-induced genes was obtained from dataset GSE26410 [30], where genes with an adjusted *p*-value < 0.05 and a log2FC > 1 were considered to be NF-κB-responsive genes (Supplementary Table S1).

### 2.9. NetworkAnalyst Approach to Study Enriched Pathways

Differentially expressed genes (DEGs) from the dataset GSE56469, comparing wild-type and PTEN KO prostate epithelial cells after laser-microdissection [47], were uploaded onto the NetworkAnalyst web platform (https://www.networkanalyst.ca [48]) (accessed on 24 April 2022) and compared with the protein interaction network of the STRING database using a cut-off of 900 with experimental validation to generate a minimum network linking all DEGs (designated as seed genes). This resulted in a network of 1008 nodes comprising 511 seeds and 2227 edges representing the links between the nodes. Upregulated nodes of this network were queried against the Reactome and the KEGG database. Using the function explorer, enriched pathways or biological functions were computed.

### 2.10. Mathematical Simulation of a Dynamic Network of AR, NF-κB, IκB, and c-Myc

In order to simulate the activities of the transcription factors AR, c-Myc, and NF-κB (p65) over time, we established a mathematical dynamic network model using *MatLab*, where we consider a six-node network including the crucial NF-κB feedback inhibitor IκB, the kinase PI3K and its inhibitor PTEN. This network was defined as follows: *i* = 1, 2, 3, 4, 5, 6 associated with AR (*i* = 1), PI3K (*i* = 2), PTEN (*i* = 3), p65 NF-κB (*i* = 4), IκB (*i* = 5), and c-Myc (*i* = 6). We use a differential equation approach that is “almost” linear [49,50], in the sense that we use terms up to first order in *x* for computing changes in the protein activities *x_i_*.

The differential equation DGL reads:

(1) $$\frac{d}{dt}\;x_{i}=J_{i}-d_{i}x_{i}+\overset{6}{\underset{j=1}{\sum }}A_{ij}x_{j}+\sigma_{i}$$

It is not the equations but the boundary condition (positivity condition), *x_i_* ≥ 0 for all *i* = 1, 2, 3, 4, 5, 6 that can give the dynamics a nonlinear character. The positivity condition stops *x_i_ at x_i_* = 0, if *x_i_* tries to become negative until it tries to grow again and can again attain positive values according to the DGL. However, it turned out that the model we consider has a fixed point x^*^ with all x_i_* > 0, so that the positivity condition is not strictly necessary for computing the fixed point of this simple system. We use the constant terms *J* = [1.5 2 −0.1 2 −0.1 0.8]; decay rates *d* = (1, 1, 1, 1), and an interaction matrix:

(2) $$A_{ij}=\left(\begin{array}{cccccc} 0 & -1 & 0 & 0.5 & 0 & 0.7\\ 0 & 0 & -2 & 1 & 0 & -0.5\\ 0 & 2 & 0 & 0 & 0 & 0\\ -0.9 & 1.5 & 0 & 0 & -3 & 0.5\\ 0 & 0 & 0 & 3 & 0 & 0\\ 0 & 0.5 & 0 & 1 & 0 & 0 \end{array}\right)$$

The constant terms *J_i_* can be thought of as base-level activity of the protein translation process that can be enhanced or suppressed by other proteins, which is encoded in the interaction matrix *A*. The decay rates *d* describe how quickly proteins are degraded again. We assume that all proteins are approximately degraded at the same rate. We also added a small noise term *σ_i_* randomly distributed between ±0.01 and we use an initial condition *x*(0) = [1 1.2 1.4 1.6 1.8 2]. We use a simple Newton-forward algorithm to integrate the equation with a time increment of *dt* = 0.001 from *t* = 0 to *t* = 150. At time *t* = 50 we made a switch of J_1 by a factor 1/2 to 0.75 (AR ablation); at *t* = 100 a switch of J_4 by a factor 1/2 to 1 (NF-κB inhibition) and at *t*=150 a switch of J_2 by a factor 1/2 to 1 (PI3K inhibition). 

Note: It should be noted that if *x(t)* is the dynamics of a system described by (*J*,*d*,*A*), we can scale the dynamics to *y_i_(t)* = *λ_i_x_i_(t)* by transforming (*J’*,*d’*,*A’*) with *J’_I_* = *λ_i_J_i_*, *d’_i_* = *λ_i_d_i_*, and *A’_ij_* = *λ_i_A_ij_λ_j_* ^−1^.

### 2.11. Statistics

Statistical analysis was carried out using GraphPad Prism 6.0 software with n-values and tests, as indicated in figure legends. The violin plots were created with ggplot2 [51]. In this case, comparison between the different group means was carried out using the Wilcox test method of the ggpubr package (v0.4.0 [52]).

## 3. Results

### 3.1. Activation of the Androgen Receptor Pathway Leads to a Downregulation of NF-κB Signaling

**Figure 2:**
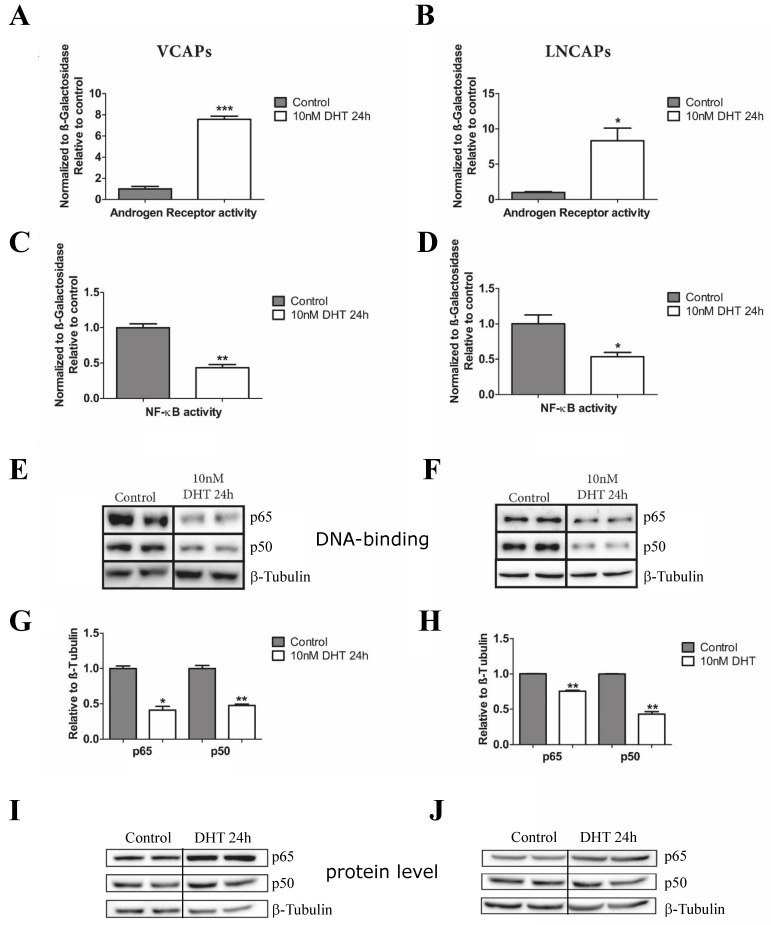
Activation of the androgen receptor leads to a downregulation of the NF-κB activity in VCAP and LNCAP cells. (**A**–**D**) Luciferase assays for control (androgen-depleted) and DHT-treated VCAP and LNCAP cells using reporter plasmids for androgen receptor (AR) as well as NF-κB transcriptional activity. (**E**,**F**) DNA binding activity of NF-κB assessed by ABCD assays as alternative to EMSAs. β-tubulin was used as a loading control. (**G**,**H**) Quantification of (**E**,**F**). Statistical analysis was performed by two-tailed Fisher’s exact test (*n* = 5; error bars represent SEM, * *p* < 0.05; ** *p* < 0.01, *** *p* < 0.001). (**I**,**J**) Protein levels of p65 and p50 NF-κB forms of samples shown in (**E**,**F**). The uncropped Western blots have been shown in Figure S5.

First, we set out to determine the interdependencies of AR activation and the NF-κB pathway in the androgen-dependent prostate cancer cell lines VCAP and LNCAPs. To that end, we depleted the cells from androgens by growing them for three days in charcoal-dextran-treated medium (which significantly reduces the level of androgens) and transfected them with luciferase reporter gene plasmids for AR-, as well as NF-κB activity. The cells were either left untreated (representing an androgen-deprived state) or incubated for 24 h in presence of dihydrotestosterone (DHT), followed by cell lysis and measurement of luciferase activity. As expected, we observed a significant increase of AR activity in both LNCAP and VCAP cells after DHT treatment (Figure 2).

In parallel, we detected a significant downregulation of NF-κB activity in both cell lines analyzed. Next, we determined DNA-binding activity of NF-κB using an ABCD assay (avidin–biotin complex with DNA, substituting classical electrophoretic mobility shift assays) with an oligonucleotide harboring four NF-κB consensus binding sites. In line with a reduced transcriptional activity of NF-κB, we found a significant reduction of p65- and p50-NF-κB protein bound to the NF-κB specific oligonucleotide after DHT treatment compared to control cells (Figure 2E–H), while the total protein levels of p65 and p50 were even increased under these conditions (Figure 2I,J). Quantitative PCR analysis of mRNA expression of several known NF-κB target genes confirmed that and revealed downregulation of the AR transcript itself, implying a negative transcriptional feedback loop (Supplementary Figure S1). Altogether, these experiments confirm that an activation of AR signaling is accompanied by a downregulation of NF-κB activity.

### 3.2. Treatment of LNCaP and VCaP Cells with Antiandrogens Leads to an Upregulation of NF-κB Signaling and a Reduction of Cell Death

**Figure 3:**
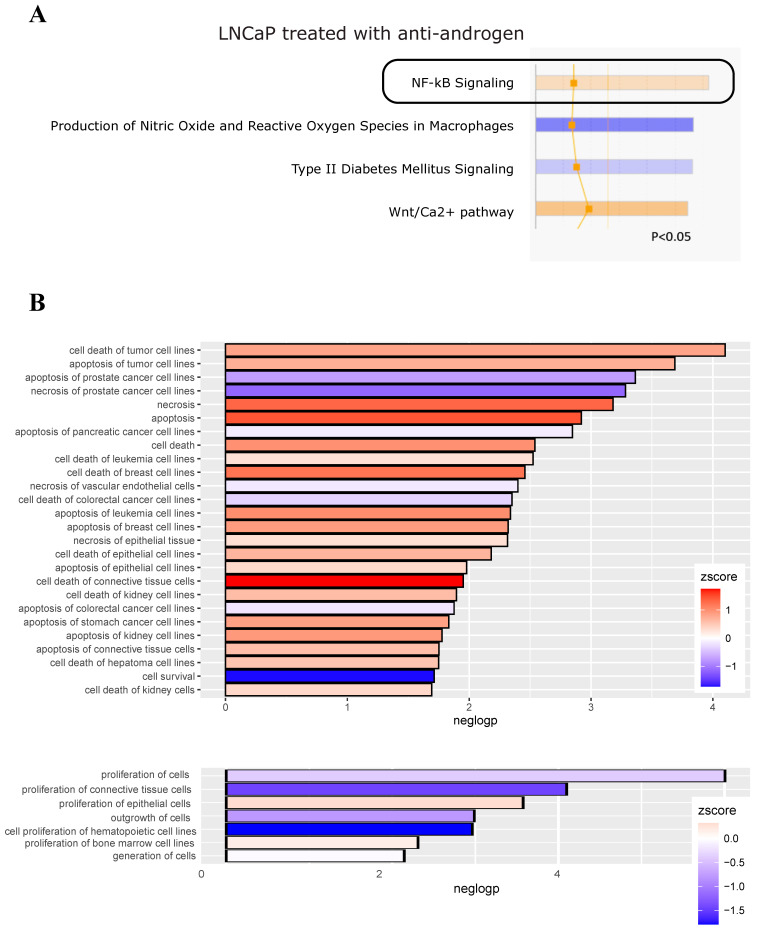
Antiandrogen treatment of LNCaP cells upregulates NF-κB signaling and reduces cell death of prostate cancer cells. A public microarray dataset of LNCaP cells treated with the antiandrogen bicalutamide (GEO database, GSE56188) was reanalyzed with the Ingenuity Pathway Analysis software. (**A**) Depiction of canonical pathways, showing NF-κB signaling significantly enhanced (orange color reflects a positive z-score, indicated by a black box). (**B**) Disease/function analysis implying that apoptosis, as well as necrosis, of prostate cancer cell lines is reduced, as reflected by the blue color (indicating a negative activation z-score), while cell death of most other cells is elevated. Lower panel: effect on proliferation of cells. Significantly altered biological functions are plotted (using the ggplot2 package of R Bioconductor) with negative log-*p*-values on the *x*-axis.

**Figure 4:**
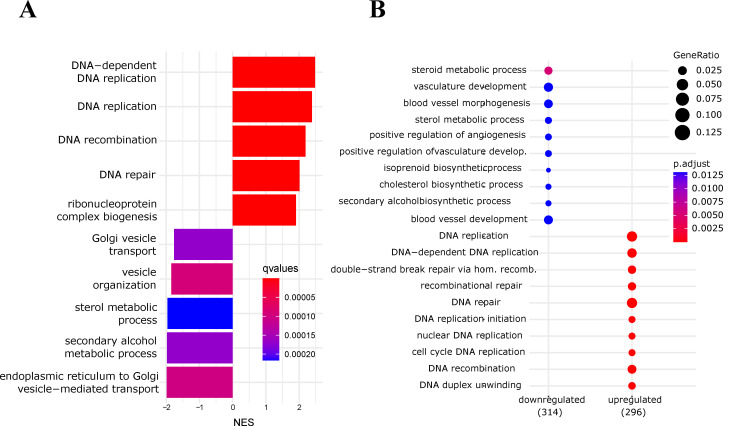
Antiandrogen treatment of VCaP cells upregulates DNA replication and reduces ER-Golgi transport processes. (**A**) Gene set enrichment analysis of VCaP cells treated with enzalutamide (GEO database, GSE62473): normalized enrichment scores (NES) of the most significant biological processes (Gene Ontology-BP) are shown. (**B**) Overrepresentation analysis (ORA) of VCaP cells treated with enzalutamide as before: most significant GO/biological processes.

Following our experimental findings that the androgen signaling axis affects the activity of the NF-κB pathway, we aimed for an unbiased bioinformatics analysis of gene regulations and corresponding biological functions. For this purpose, we reanalyzed several publicly available transcriptomics datasets of LNCaP and VCaP cells. The first one (GSE56188) was generated with LNCaP cells treated with the common antiandrogen bicalutamide (which is considered a second-generation antiandrogen). *Ingenuity Pathway Analysis* with the commercial software from Qiagen implied an upregulation of NF-κB signaling, as assessed by a positive z-score. Furthermore, prediction of effects on biological functions indicated that both apoptosis and necrosis of prostate cancer cells are downregulated, while death of most other cell types was predicted to be increased (Figure 3A,B). Analysis of VCaP cells treated with the third-generation antiandrogen enzalutamide (gene set GSE62473) revealed an upregulation of DNA replication, DNA repair, and cell cycle. Furthermore, ER-Golgi transport processes seemed downregulated, in line with a recent report of others (Figure 4A,B) [53]. Treatment of VCaP cells with bicalutamide had nearly identical consequences to treatment with enzalutamide (Supplementary Figure S2).

### 3.3. Mutual Inhibition of AR and NF-κB Signaling Is Also Evident In Vivo

**Figure 5:**
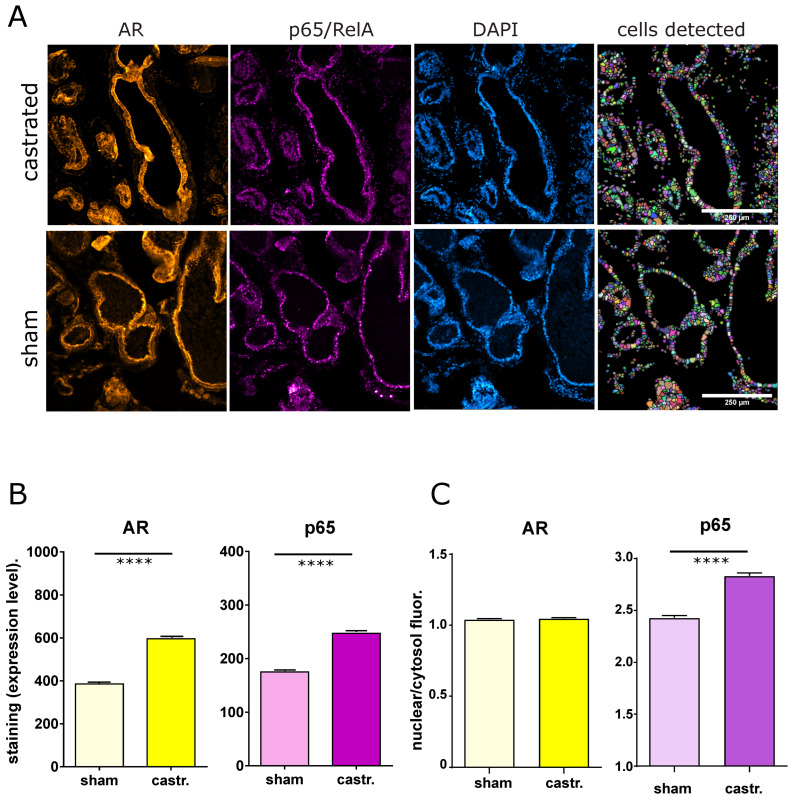
Immune-fluorescence microscopy of AR and p65 in castrated versus sham-operated mice. (**A**) Representative fluorescence microscopy images of prostate tissue sections from C57BL/6 mice (castrated or sham-operated) stained for AR, p65/RelA (NF-κB), and DAPI (for DNA and cell recognition). The right panel shows the cell masks detected with the IKOSA-image analysis platform (see Section 2) The scale bar indicates 250 µm. Samples from castrated mice (castr.) comprised 6 sections, from which 33 view fields were captured for the three fluorescence microscopy channels. Cell recognition resulted in the detection of 30,988 cells. Samples from sham-operated mice comprised 7 sections, 29 view fields, and resulted in the detection of 27,665 cells. (**B**) Quantification of cellular fluorescence intensities for AR, p65, and DAPI (as control) for sham-operated and castrated mice; median values with error bars representing the 95% confidence interval. Statistical analysis was performed with a nonparametric Mann–Whitney test (**** *p* < 0.0001). (**C**) Quantification of nuclear to cytosolic fluorescence for AR and p65 (images from A, median values with error bars representing the 95% confidence interval, **** means *p* < 0.0001).

Next, we were interested in investigating whether AR and NF-κB are also mutually inhibitory in vivo. To answer that, we first used a mouse model of androgen ablation achieved by castration and compared that with sham-operated mice. Immune-fluorescence microscopy with automated cell recognition and intensity quantification revealed an upregulation of the p65/RelA member of the NF-κB family after castration, indicating that downregulation of AR activity leads to an increase in NF-κB protein expression and presumably higher basal NF-κB activity. The latter was also supported by a higher nuclear/cytosolic ratio of NF-κB after castration, which was not observed for AR. However, castration of mice also led to an increase of the AR protein level, which seems to be a feedback mechanism trying to escape the loss of androgens (Figure 5).

**Figure 6:**
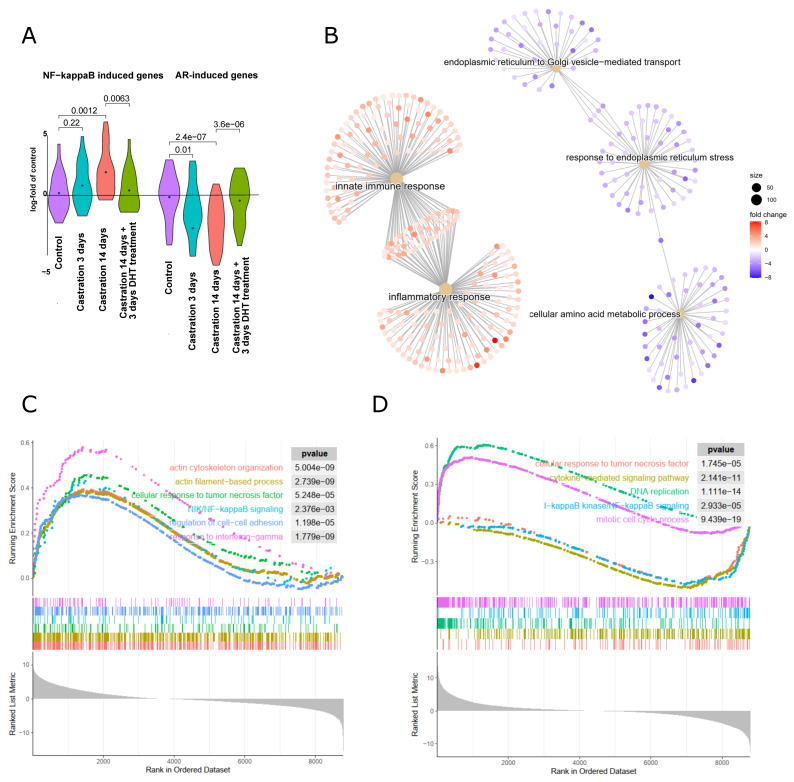
Gene expression changes in the mouse model of antiandrogen treatment via castration. (**A**) Gene expression data from prostates derived from mice that were castrated to eliminate androgen signaling, or sham-operated (control) followed by RNA extraction after 3 days or 14 days as indicated. One cohort was treated with dihydrotestosterone for 3 days, 14 days after castration to reactivate androgen signaling (castr. 14 d + DHT 3 d, dataset GEO GSE5901, [28]). Androgen-induced genes [46] and NF-κB target genes (Supplementary Table S1) are shown as violin plots in comparison to control prostates. (**B**) Comparison of biological processes in prostates of mice 3 days after castration as compared to sham-operated mice: gene ontology analysis of upregulated (red nodes) or downregulated (blue nodes) processes. (**C**) Gene set enrichment analysis (GSEA) of mice 14 days after castration as compared to sham-operated control mice. Inflammatory signaling pathways, cell adhesion, and actin-based processes are upregulated. (**D**) GSEA after readdition of testosterone to castrated mice as compared to the castrated mice: mitotic cell cycle and DNA replication are upregulated and NF-κB signaling processes (response to TNFα, cytokine-mediated signaling, and IKK/NF-κB signaling) are downregulated.

Next, we analyzed a published microarray dataset of castrated mice, where gene expression of prostate tissues was analyzed 3 days or 14 days after castration [28]. Control mice were sham-operated and analyzed 3 days afterwards. A fourth cohort of mice had been castrated, followed by implantation of a testosterone-releasing pellet after 14 days, and isolation of prostate RNA three days afterwards. Using this dataset, we checked for the activity of the AR receptor signaling axis using a list of AR-induced genes as previously published [46]. As expected, AR-induced genes were downregulated significantly after castration, and reinduced again after testosterone treatment, while the opposite was observed for NF-κB target genes (Figure 6A). As early as three days after castration, inflammatory and innate immune response genes are significantly induced, while ER-stress and ER-Golgi transport genes are downregulated in line with data obtained for cultured human prostate cancer cells (Figure 6B). These gene alterations are further aggravated 14 days after castration, as assessed by gene set enrichment analysis (GSEA), revealing a substantial induction of NF-κB and interferon-gamma signaling genes, as well as genes involved in cell adhesion and actin cytoskeletal functions, presumably reflecting leukocyte infiltration (Figure 6C). Performing this type of analysis with prostate samples of mice treated for 3 days with testosterone 14 days after castration in comparison with the castrated mice showed a downregulation of NF-κB and cytokine signaling, while DNA replication and mitosis genes were induced (Figure 6D). Altogether, these analyses imply that blockade of AR signaling results in an upregulation of NF-κB and inflammatory pathways and a concomitant downregulation of ER/Golgi-pathways. Reactivating AR activity with testosterone seems to drive cell proliferation, in line with the notion that AR blockade may hamper cell cycle progression while simultaneously activating cell survival mechanisms.

### 3.4. Long-Term Androgen Deprivation Upregulates NF-κB Activity and Inflammatory Pathways

**Figure 7:**
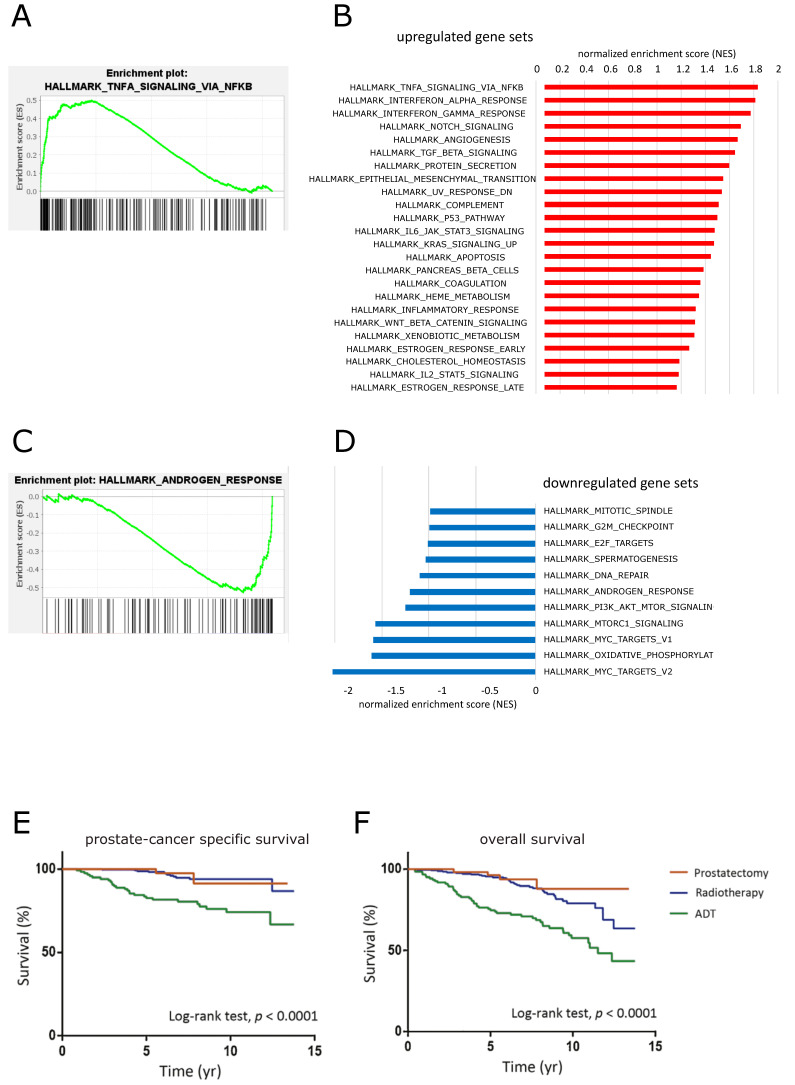
Effects of long-term androgen deprivation in human LNCaP cells and in a clinical study. Gene set enrichment analysis of genes after 5 months of androgen deprivation as compared to control cells (GSE8702, [27]). (**A**) Genes associated with TNFα-mediated NF-κB signaling are significantly enriched by androgen deprivation. (**B**) Normalized enrichments scores (NES) of hallmark gene sets significantly enriched in androgen-deprived cells, including several inflammation-response pathways (*p* < 0.01). (**C**) Androgen depletion leads to a downregulation of androgen response genes. (**D**) Hallmark gene sets were significantly enriched in control cells as compared to androgen-deprived cells (*p* < 0.01); thus, were downregulated after androgen depletion for 5 months. (**E**,**F**) Survival data of patients with metastatic or locally advanced prostate cancer treated by prostatectomy, radiotherapy, or androgen deprivation therapy (ADT), as indicated. The figure is reproduced from [54] based on a creative commons license (CC BY-NC-ND 4.0) and with permission of the original editor (Elsevier).

Since androgen-independence of prostate cancer is a feature that usually develops slowly after androgen ablation, we also analyzed long-term changes that occur in absence of androgens. For that purpose, we investigated a dataset (GSE8702) where LNCAP cells were starved in androgen-depleted medium for five months in comparison to control cells [27]. Using gene set enrichment analysis (GSEA), we could observe a significant upregulation of the NF-κB signaling axis after androgen starvation in parallel with a reduction of genes responding to androgen (Figure 7). In line with that, AR-repressed genes were significantly induced (Supplementary Figure S3). Apart from that, gene set analysis revealed the upregulation of several inflammation associated pathways (such as interferon, IL-6, or complement pathways), as well as an enrichment of genes involved in epithelial–mesenchymal transition. Biological processes predicted to be downregulated included various cell cycle or proliferation pathways, as well as DNA repair. These data support the notion that androgen depletion might reduce the proliferative potential of prostate epithelial cells at least in vitro, while simultaneously upregulating inflammatory pathways.

These cellular consequences of androgen deprivation are in line with a published clinical study of patients suffering from advanced prostate cancer [54], which implied that long-term androgen deprivation therapy is actually resulting in a worse prognosis as compared to nonhormone targeting therapies such as prostatectomy and radiotherapy (Figure 7E,F).

### 3.5. Loss of PTEN Upregulates NF-κB Signaling as Well

**Figure 8:**
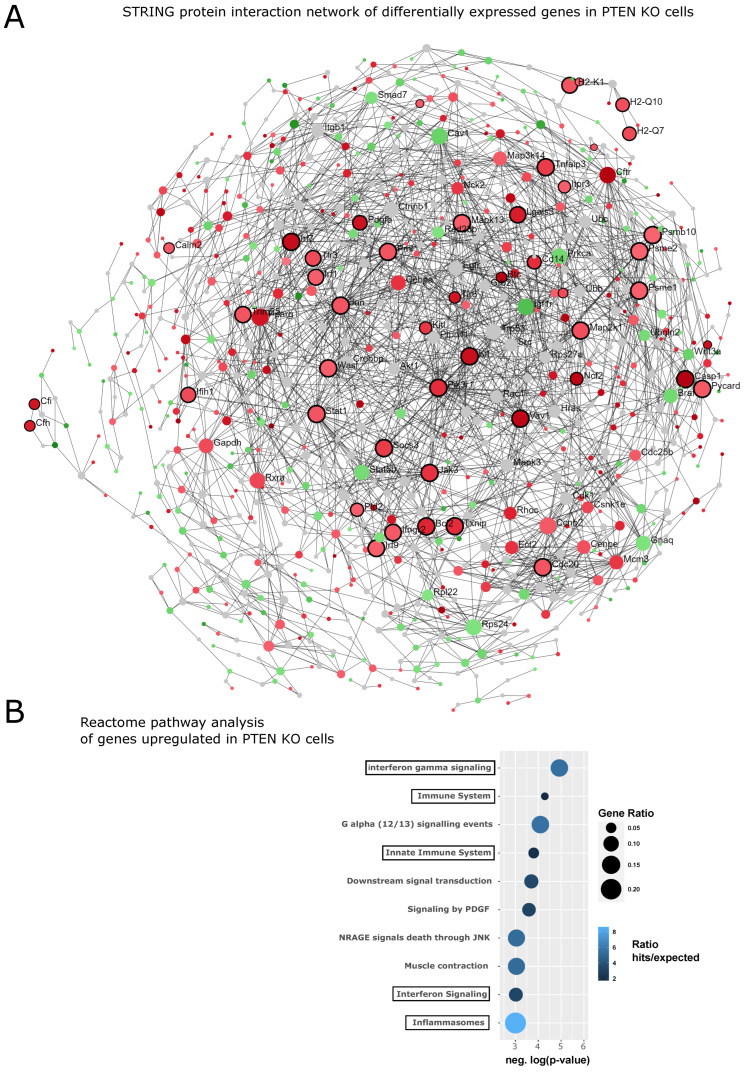
Loss of PTEN activates NF-κB and inflammatory pathways. (**A**) A dataset of WT- and PTEN-deficient prostate epithelial cells from a mouse model of prostate cancer [47] was reanalyzed on the NetworkAnalyst platform by comparing differentially expressed genes with the protein interactome of the STRING database and computing a minimal joint network. Upregulated nodes (shown in red) were then queried against the Reactome database to elucidate functional enrichments. Inflammatory pathways were selected, which are highlighted by black borders of the respective nodes. (**B**) Overview of the most significantly enriched Reactome pathways among upregulated genes. Black frames around the pathway terms are those reflecting the black borders in (**A**). Negative log-*p*-values are plotted in such a way that the bubble size represents the gene ratio (regulated genes/total pathway genes) and the color reflects the ratio of gene hits per expected.

Loss of PTEN function, which leads to activation of the PI3K/Akt-pathway, is found in about 20% of primary prostate cancers and in about 50% of androgen-independent tumors, indicating that it may also contribute to the acquisition of castration resistance [46,55]. Indeed, counter-regulation of PI3K and AR signaling has been found in prostate cancer, where PTEN deletion reduces AR activity and inhibition of AR results in activation of PI3K/Akt signaling [29]. Furthermore, the PTEN/PI3K/Akt axis is linked to NF-κB activation, as Akt can phosphorylate IKK1 (IKKα), a component of the IKK complex, which is activating NF-κB [56]. Therefore, it is plausible to assume that reduction of PTEN expression might also lead to an activation of NF-κB and thus an inflammatory state. This has been demonstrated, for instance, for leukemia cells [57] and was also recently suggested for a mouse model of PTEN-deficiency-associated prostate cancer [58]. To investigate this notion by bioinformatic means, we analyzed two datasets of murine prostates with deletion of PTEN. The first used laser microdissection of benign or cancerous PTEN-KO prostate epithelial cells (from 28–30 weeks old mice) and a whole-mouse genome microarray to decipher differences in gene expression [47]. The second used the same PTEN KO mouse model (Pb-Cre/PTEN^lox/lox^) and analyzed the RNA from whole prostates of eight-week-old mice [29]. Reanalysis of the epithelial-cell-specific first dataset was performed via the NetworkAnalyst platform [48] by uploading differentially expressed genes (using an adjusted *p*-value < 0.05 as threshold), followed by comparison with the STRING database interactome and generation of a minimum network linking all the seed genes (n = 511). This resulted in a network of 1008 nodes and 2227 edges, which was then queried against the Reactome database to identify enriched pathways (Figure 8). Inflammatory and immune defense pathways were significantly enriched for upregulated genes, supporting the notion that loss of PTEN induces inflammatory signaling. Similar results were obtained for the second dataset, revealing an upregulation of NF-κB target genes after deletion of PTEN, which was further enhanced by castration and, thus, androgen deprivation (Supplementary Figure S4).

### 3.6. Mathematical Model of a Dynamic AR/NF-κB/c-Myc Network

**Figure 9:**
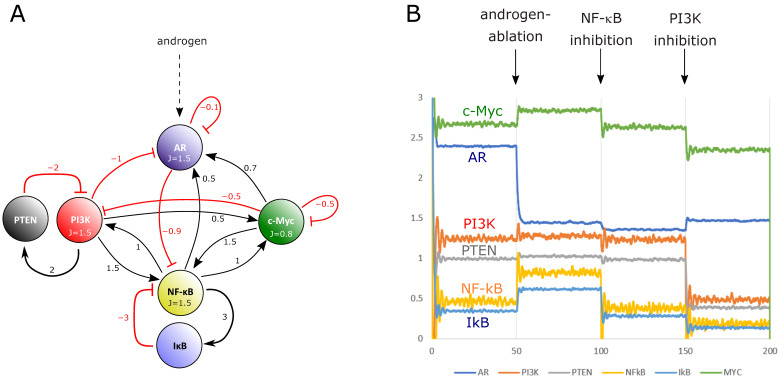
Mathematical simulation of a dynamic network between AR, NF-κB/IκB, and c-Myc. (**A**) Interdependent network between AR, NF-κB/IκB, PI3K/PTEN, and c-Myc with estimated activating or inhibitory links as indicated by the numbers of lines linking the nodes. The presence of androgens provides a stimulatory effect on AR. (**B**) Time course of activities (arbitrary units) as predicted by the model. Loss of androgens or inhibition of AR (androgen-ablation arrow) leads to upregulation of NF-κB and c-Myc activities, while subsequent inhibition of NF-κB and PI3K results in a downregulation of c-Myc activity below the original level.

Transcription factors often build dynamic gene-regulatory networks with mutual positive or negative feedbacks. This behavior can be simulated mathematically using differential equations to compute dynamic networks [59]. We applied that concept to the mutual influences of AR and NF-κB including the oncogene c-Myc, which is crucial in a high percentage of prostate cancers. Furthermore, we considered IκB, the main inhibitor of NF-κB, which is transcriptionally upregulated by NF-κB, thereby providing a negative feedback loop for this central transcription factor of inflammation. In addition, we implemented the crucial tumor suppressor PTEN, which is lost in a high percentage of prostate cancer cases, and its downstream signaling kinase PI3K. A dynamic interdependence network was constructed with activating or inhibitory links, based on prior experimental data obtained with reporter gene assays or with gene expression datasets (Figure 9A). Using the mathematical approach described in the Methods section, we computed steady-state activity levels of the network nodes. Following that, we perturbed the system by simulating androgen ablation, as is clinically performed by androgen receptor antagonists to treat metastasizing prostate cancer. In line with our experimental data and bioinformatic analyses, the mathematical model predicted an upregulation of NF-κB activity. Interestingly, c-Myc activity increased in the simulation as well, implying that androgen deprivation might elevate potential effects of this crucial oncogene. However, a subsequent reduction of NF-κB activity (e.g., using anti-inflammatory drugs or inhibitors of NF-κB activating enzymes) results in a downregulation of both NF-κB and c-Myc activity in the model, with the latter even slightly below the level it had before androgen ablation. Further inhibition of PI3K resulted in an even more pronounced downregulation of the predicted c-Myc activity (Figure 9B). This indicates that anti-inflammatory therapeutics given together with androgen receptor antagonists, and probably additional PI3K inhibitors, might have a beneficial effect for the therapy of metastasizing prostate cancer.

## 4. Discussion

Androgen deprivation, either by interfering with synthesis of testosterone or by blocking androgen receptor (AR) activity with AR antagonists such as bicalutamide or enzalutamide, is still the standard therapy of metastasizing prostate cancer. However, basically all patients with this form of advanced prostate cancer suffer from a tumor relapse after androgen deprivation and an initial decline of the tumor burden. We hypothesized that blockade of androgen receptor function, which is a crucial differentiation factor of prostate epithelial cells, might increase the cellular stress so that the NF-κB signaling pathway might be activated as one of the central defense and stress response pathways. We could confirm a counter-regulatory link between AR and NF-κB signaling both experimentally (via reporter gene and DNA binding assays) and by computational means and meta-analysis of a variety of datasets. Since NF-κB upregulates several important antiapoptotic genes, it seems plausible that an increase in NF-κB activity after antiandrogen treatment actually provides a survival advantage to metastasizing prostate cancer cells. This hypothesis is supported by pathway analyses implying an upregulation of NF-κB signaling with a concomitant reduction of apoptosis and necrosis specifically in prostate cancer cells, while cell death is rather increased in other cells. Although the investigated AR-dependent human prostate cancer cell lines (LNCaP and VCaP) vary a bit in their response to AR antagonists, both of them do not show any significant increase of cell death upon treatment. These findings were supported by in vivo mouse models of prostate cancer and castration to cause androgen deprivation. NF-κB induced genes were elevated after castration and reduced again after readdition of testosterone and the opposite was observed for AR-induced genes. Again, pathway analysis implicated an increase of inflammation and a downregulation of ER-Golgi processes, which was confirmed by gene set enrichment analyses. The latter also supported the notion that NF-κB signaling is downregulated after readdition of testosterone to castrated mice in line with a counterbalance of AR- and NF-κB activity. This concept was also supported by a dataset, where LNCaP cells were rendered AR-independent by long-term cultivation in the absence of androgens (to simulate long-term treatment of patients with antiandrogens). Under these conditions, NF-κB and inflammatory signaling, as well as epithelial–mesenchymal transition, were significantly enhanced in parallel with a reduction of the androgen response. Although cell cycle processes were also predicted to be downregulated under these conditions, these data still support the notion that AR blockade fails to eliminate AR-dependent prostate cancer cells. Similar observations were made in a more recent clinical study of patients with metastasizing or locally advanced prostate cancer treated with prostatectomy, radiotherapy, or androgen deprivation therapy [54]. Here, patients with androgen deprivation exhibited the fastest decrease of the survival probability (see Figure 7E,F).

While it seems clear that loss of androgen function results in an upregulation of NF-κB and inflammatory signaling, the interdependence in the other direction seems different. Here, an induction of NF-κB activity rather leads to an elevation of AR activity, which coined the concept of a positive cooperativity between NF-κB and AR and might be interpreted as protumorigenic effect of AR [20,21]. However, this could also mean that the cells try to evade the effect of NF-κB by upregulating AR (which causes a feedback inhibition of NF-κB). Recent observations indicate that AR function is somewhat lost at early prostate cancer stages and regained in a pathological manner at later stages. The normal androgen receptor has been shown to act as differentiation factor for prostate epithelial cells rather than as a transforming factor, at least at the early phase of tumorigenesis [60,61]. At later stages of cancer development, epigenetic changes seem to reprogram the AR transcriptome, presumably by activating developmental processes and AR binding sites that have a role in prostate organogenesis [62,63].

Interestingly, loss of PTEN, one of the most frequent oncogenic alterations in the pathogenesis of prostate cancer, leads to an upregulation of NF-κB and inflammatory signaling as well. This indicates that deficiency of this crucial tumor suppressor might act cooperatively with therapeutic AR blockade to upregulate stress response pathways, which finally activate survival mechanisms of cancer cells via NF-κB-mediated induction of antiapoptotic genes. While the available data do not directly or mechanistically prove a causative role of elevated NF-κB activity in prostate cancer cells in therapy resistance or tumor progression, our combined experimental and computational analyses strongly suggest that androgen deprivation results in upregulation of NF-κB activity, which has been demonstrated in numerous studies to have a protumorigenic role.

A differential-equation-based mathematical model of the dynamic interdependencies between AR, c-Myc, PI3K/PTEN, and NF-κB/IκB actually predicts the upregulation of NF-κB activity after inhibition of AR—and, strikingly, also an increase of the activity of the oncogene c-Myc. Subsequent inhibition of NF-κB in this model leads to reduction of c-Myc slightly below the original activity level, while AR activity is not significantly altered anymore. Additional inhibition of PI3K results in a further reduction of c-Myc activity in the model and a further downregulation of NF-κB. It is evident that such a six-component network cannot reflect the complexity of the cancer situation in vivo. Nevertheless, it confirms our experimental and computational analyses and implies that the dogma of antiandrogen therapy of prostate cancer ought to be reconsidered carefully and that new therapeutic approaches, presumably applying combination therapies, should be sought for metastasizing prostate cancer, which represents the second-leading cause of cancer-related death in men.

Based on our observation we think that it would be important to test combined AR- and NF-κB inhibition, probably including PI3K inhibitors, in preclinical prostate cancer models, and at a later stage also in clinical studies of metastasizing prostate cancer. In principle, inflammatory NF-κB signaling might be inhibited by commonly used anti-inflammatory drugs such as glucocorticoids (e.g., dexamethasone), but we assume that this would be too unspecific due to further effects on the glucocorticoid receptor. Therefore, we expect that a more specific inhibition of NF-κB signaling with inhibitors of IκB kinases (IKK1 and IKK2) might be superior. Furthermore, different regimens of NF-κB blockade in combination with AR antagonists (and PI3K inhibitors) would have to be tested, such as continuous versus intermittent. The latter might have advantages by allowing a recovery of the (NF-κB-dependent) immune system in between AR/NF-κB blockade phases, to improve immune defense against cancer cells.

## 5. Conclusions

We conclude that several lines of evidence support a counter-regulatory link between androgen receptor and NF-κB signaling and that therapeutic downregulation of AR activity by androgen ablation or AR antagonists causes an upregulation of NF-κB signaling, representing a general stress response pathway. Since antiapoptotic genes belong to the target genes of NF-κB, this implies a potential survival advantage for metastasizing prostate cancer cells caused by the treatment leading to the notion that a combined anti-AR/anti-NF-κB treatment might outperform the standard antiandrogen treatment.

## Data Availability

Data are available from the corresponding author upon reasonable request.

## Supplementary Materials

The following are available online at https://www.mdpi.com/article/10.3390/cancers14246164/s1, Figure S1: Quantitative PCR analysis of mRNA expression after DHT treatment, Figure S2: Computational analysis of VCaP cells treated with bicalutamide (GSE62473), Figure S3: Gene set enrichment analysis (GSEA) of LNCaPs androgen-deprived for 5 months [46], Figure S4: Increase of NFκB target gene expression by loss of androgen and of PTEN function [29,30], Figure S5: The uncropped Western blots, Table S1. NF-κB responsive genes from GSE26410.
