# Supplementary material for: Antagonistic Functions of Androgen Receptor and NF-κB in Prostate Cancer—Experimental and Computational Analyses
Source: Cancers (Basel). 2022 Dec 14;14(24):6164. doi: 10.3390/cancers14246164 (PMC9776608; doi:10.3390/cancers14246164)
Supplement: Supplementary file 1 [file cancers-14-06164-s001.zip › cancers-1908083-supplementary.pdf]

cancers

Supplementary Materials: Antagonistic Functions of Androgen Receptor and NF-κB in Prostate Cancer—Experimental and Computational Analyses

José Basílio, Bernhard Hochreiter, Bastian Hoesel, Emira Sheshori, Marion Mussbacher, Rudolf Hanel and Johannes A. Schmid [1,*]

1. Supplementary methods:

1.1. RNA extraction and qPCR analysis

RNA was isolated using Qiazol reagent (Qiagen), according to the manufacturer´s instructions. For qPCR analysis cDNA was generated using RevertAid First Strand cDNA synthesis Kit (Thermo Scientific) according to the manufacturer´s instructions. qPCR was performed using SYBR Green (Thermo Scientific) or TaqMan Reagents (Thermo Scientific). Primers for qPCR analysis were as follows:

c-myc_for: AGCGACTCTGAGGAGGAACA,
c-myc_rev: CTCTGA-CCTTTTGCCAGGAG,
GAPDH_for: CCTGTTCGACAGTCAGCCG,
GAPDH_rev: CGACCAAATCCGTTGACTCC,
cxcl5_for: ATTTTGGACGGTGGAAACAA,
cxcl5_rev: TGTCTTCCCTGGGTTCAGAG,
cxcl12_for: TCAGCCTGAGCTACAGAT-GC,
cxcl12_rev: CTTTAGCTTCGGGTCAATGC,
AR_for: GTGGAAGCTGCAAGGT-CTTC,
AR_rev: GGCGCACAGGTACTTCTGTT,
ERG_for: CAAGTAGCCGCCT-TGCAAA,
ERG_rev: GCTCCAGGAGGAACTGCCA,
ERG_probe: FAM-CCAGGCAGTGGCCAGATCCAGC-TAMRA,

2. Supplementary figures:

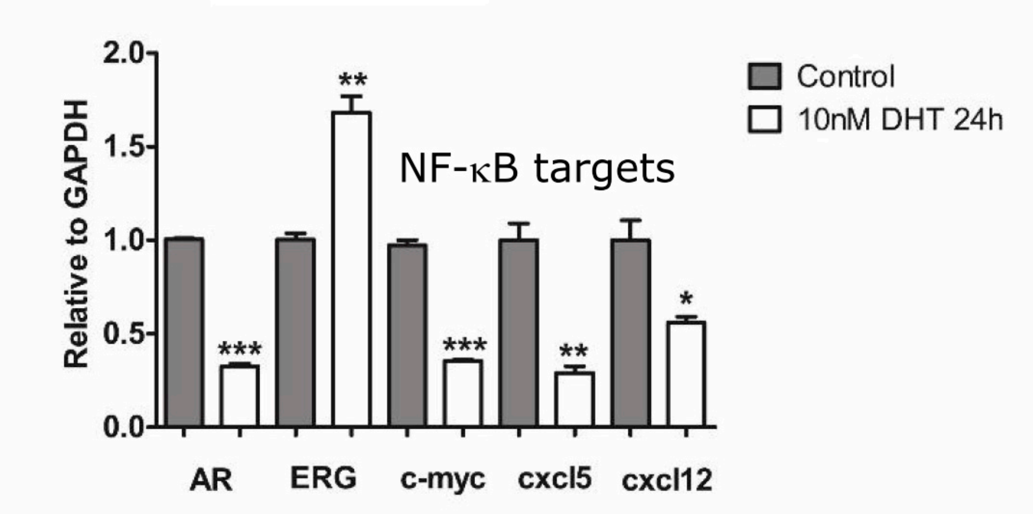

Figure S1. Quantitative PCR analysis of mRNA expression after DHT treatment. VCaP cells (which contain a gene fusion placing the Ets related gene ERG downstream of the androgen-dependent TMPRSS2 promoter) were treated with dihydro-testosterone (DHT) for 24h, followed by RNA extraction and quantitative RT-PCR. This revealed a downregulation of known NF-κB target genes (c-myc, cxcl5 and cxcl12) after DHT-treatment, as well as a reduction of the AR mRNA itself (implying

a negative feedback loop), while ERG expression was upregulated. (n = 3, error bars represent SEM, *$p < 0.05$; **$p < 0.01$, ***$p < 0.001$).

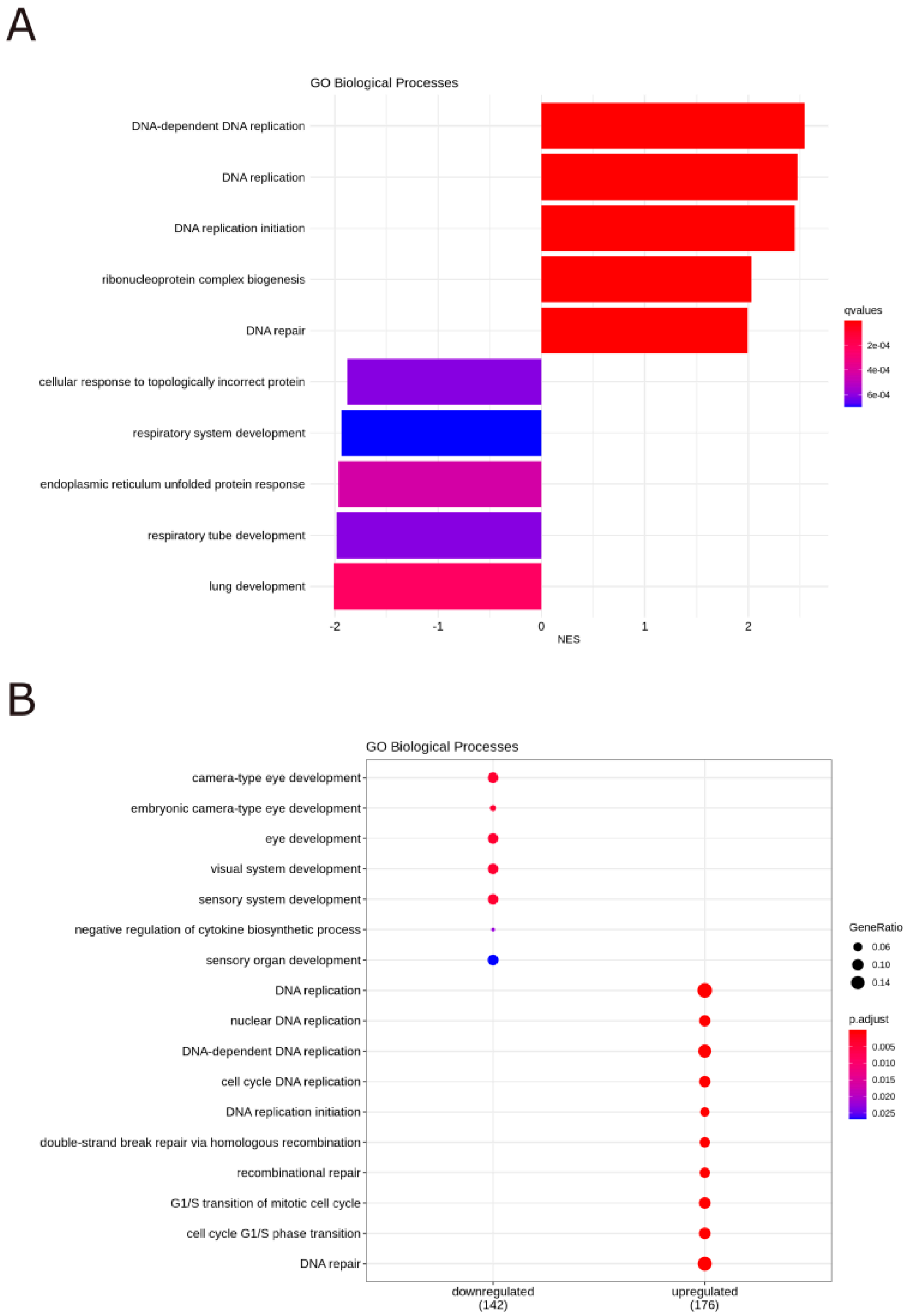

Figure S2. Computational analysis of VCaP cells treated with bicalutamide (GSE62473). **(**A) Gene set enrichment analysis: most significant Gene Ontology/Biological Processes**;** (B) Over-representation analysis (ORA) of the most significant biological processes.

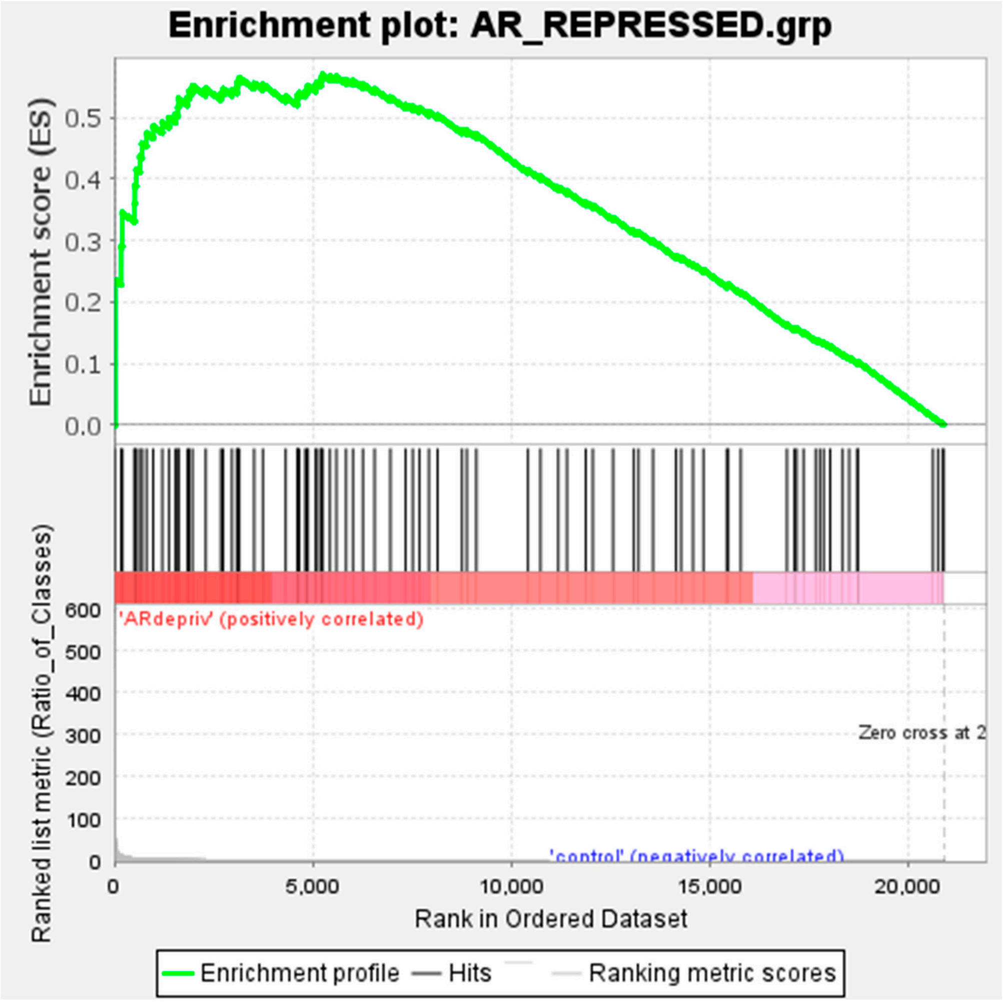

Figure S3. Gene set enrichment analysis (GSEA) of LNCaPs androgen-deprived for 5 months. Gene set GSE8702 was analyzed (2 samples of 5 months deprivation versus 2 controls: 0 and 3 weeks) using the GSEA software of the Broad Institute with a gene set of AR-repressed genes published in [46] Mulholland DJ, et al., Cancer Cell 2011, Vol. 19: 792-804, https://doi.org/10.1016/j.ccr.2011.05.006. Genes repressed by AR are enriched with a normalized enrichment score (NES) of 1.65.

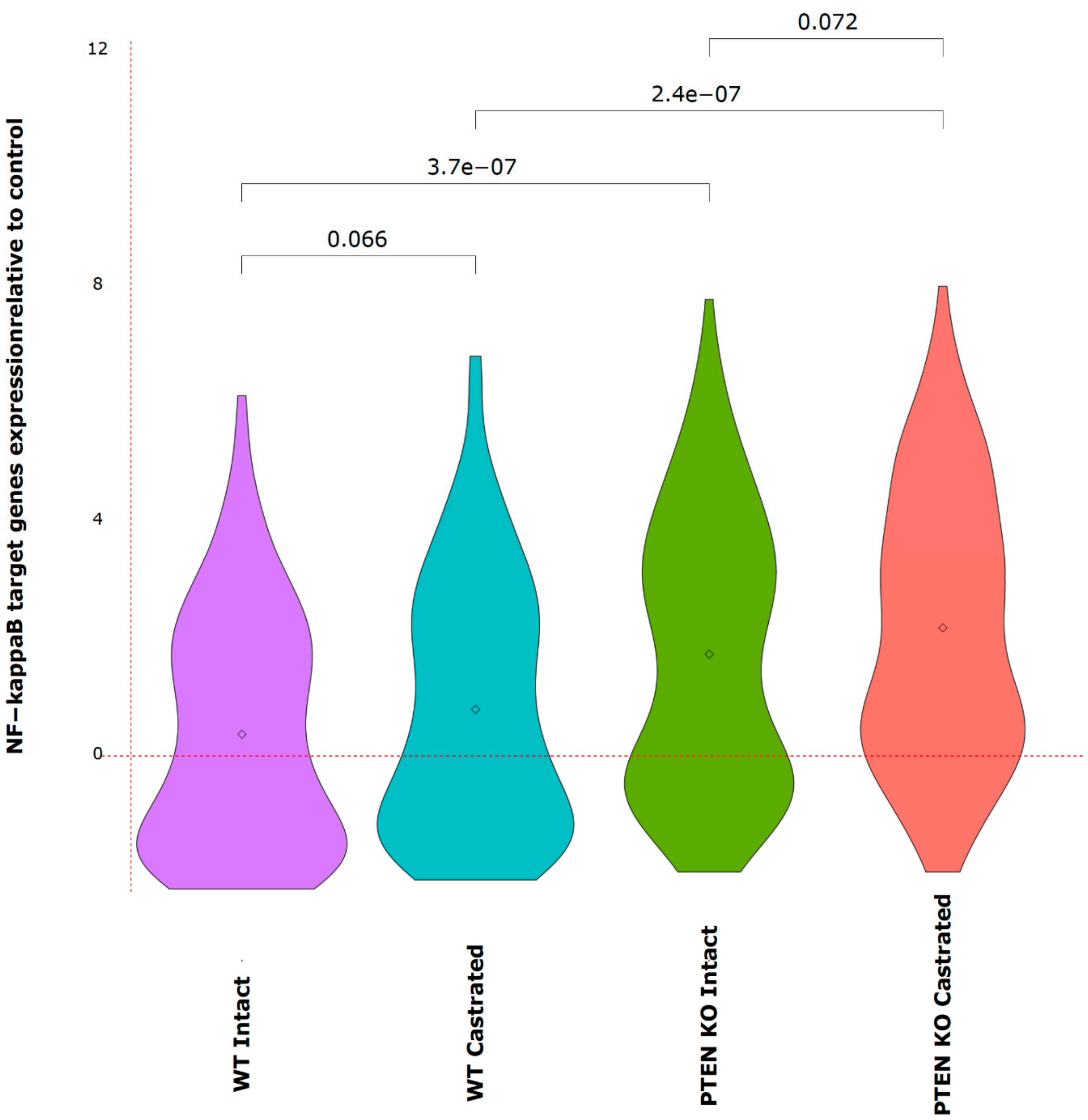

Figure S4. Increase of NFκB target gene expression by loss of androgen and of PTEN function. The dataset GSE24691 [29] (Carver et al. *Cancer Cell* 2011: https://doi.org/10.1016/j.ccr.2011.04.008) of wild-type (WT) and prostate-specific PTEN-knockout mice (PTEN KO) comprising the whole prostate including epithelial and stromal cells was analyzed for the expression of NFκB target genes (Suppl. Table 1, derived from [30] Birbach et al. *Neoplasia* 2011: https://doi.org/10.1593/neo.11524). Mice of the 2 genotypes were either left untreated or castrated and analyzed 3 days afterwards. Androgen ablation by castration led to an upregulation of NFκB target genes – and the same was observed after loss of PTEN. The highest upregulation was observed in castrated PTEN-KO mice. Bars linking the violin plots indicate the p-values of significance of the differences.

LNCaP p65.

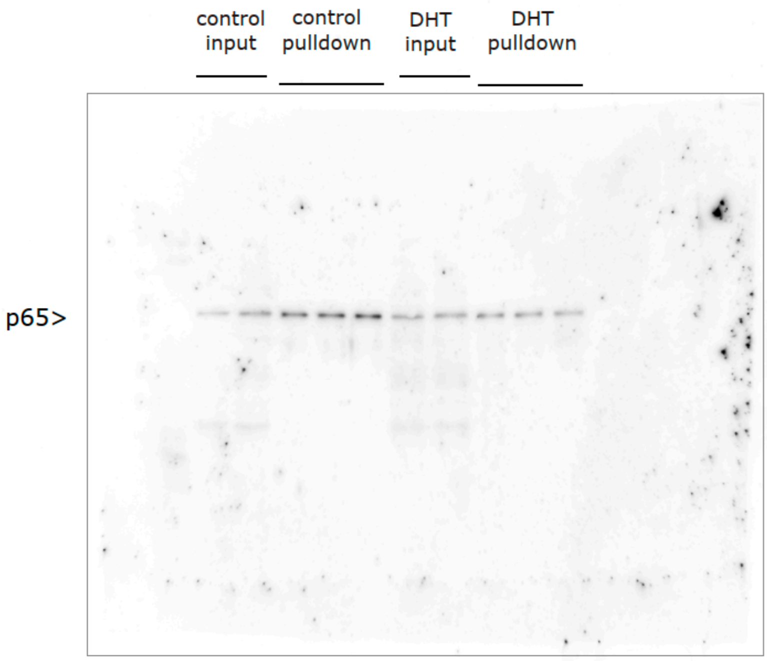

LNCaP p50.

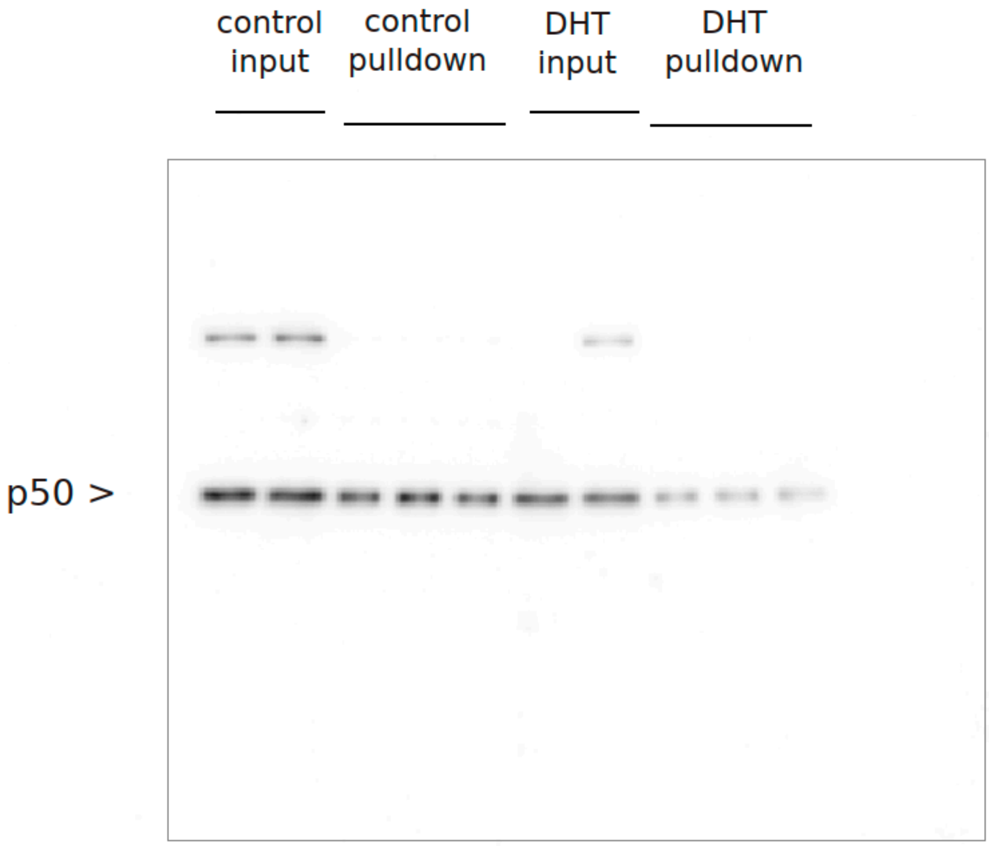

LNCaP: β-Tubulin. (new sample application).

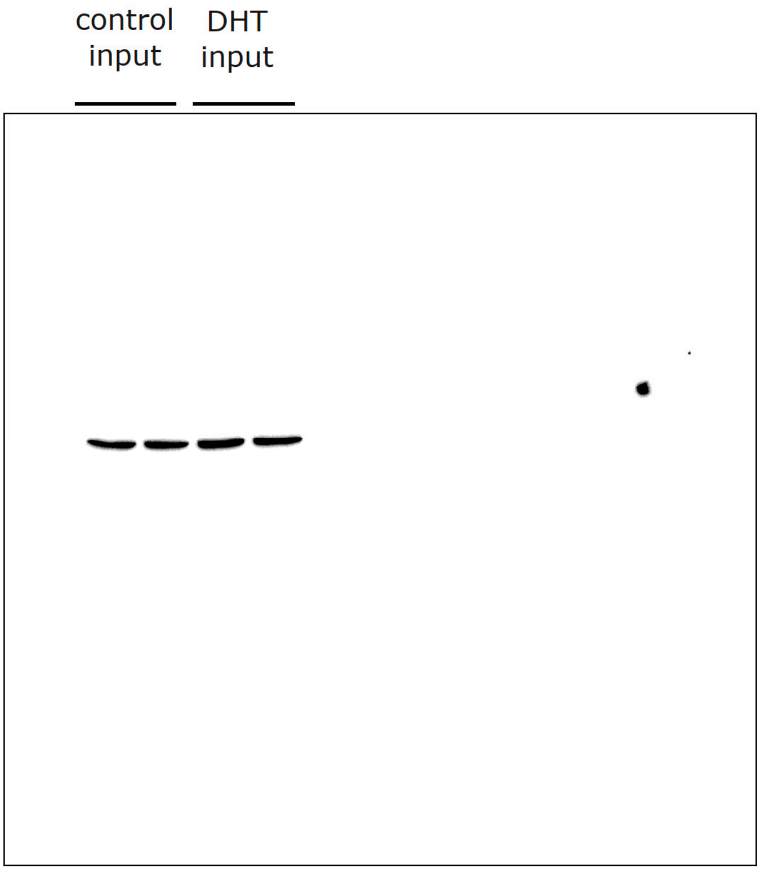

VCaP p65.

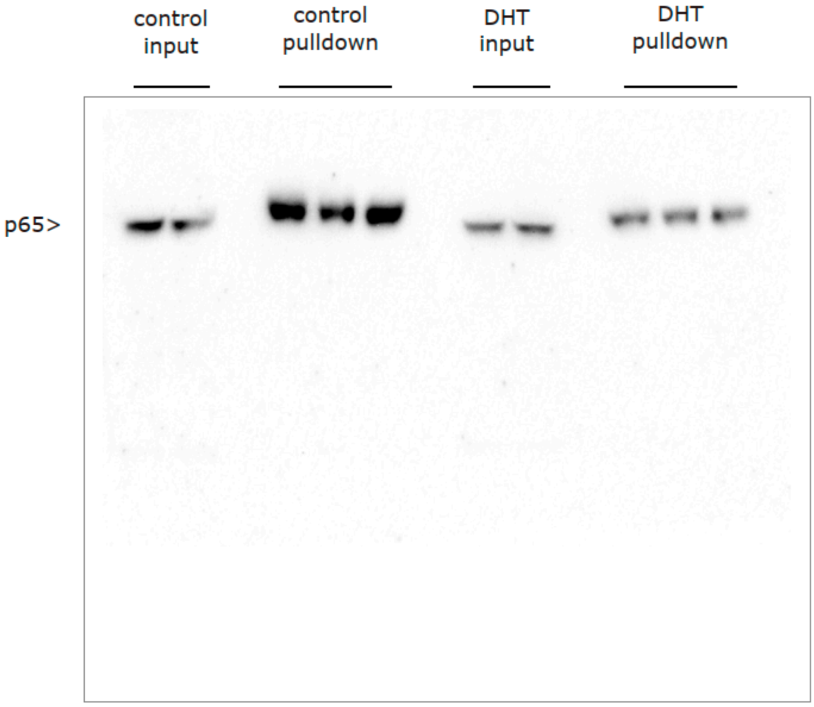

VCaP p50.

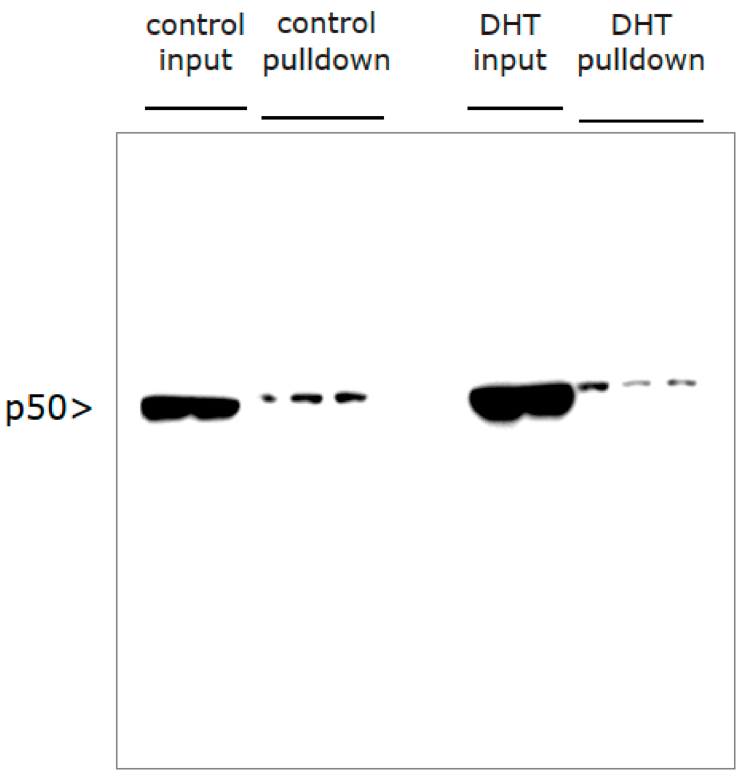

VCaP β-Tubulin.

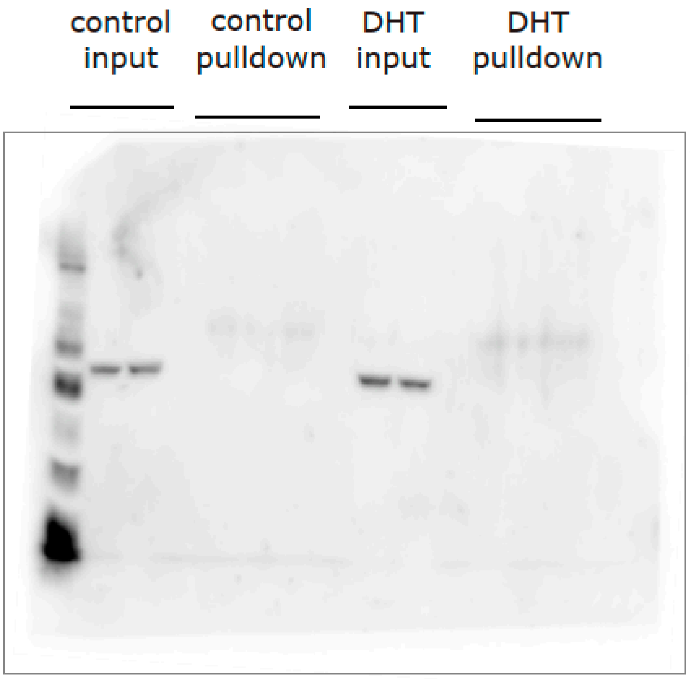

Figure S5. Full Western blots. For Figure 1 E, F, I, J.

Table S1. NF-κB responsive genes from GSE26410.

PROBEID	ENTREZID	ENSEMBL	SYMBOL	GENENAME
10449000	56047	ENSMUSG00000063011	Msln	mesothelin
10581605	15439	ENSMUSG00000031722	Hp	haptoglobin
10538732	14804	ENSMUSG00000071424	Grid2	glutamate receptor, ionotropic, delta 2
10549041	108096	ENSMUSG00000063975	Slco1a5	solute carrier organic anion transporter family, member 1a5
10425354	17309	ENSMUSG00000042428	Mgat3	mannoside acetylglucosaminyltransferase 3
10583519	15894	ENSMUSG00000037405	Icam1	intercellular adhesion molecule 1
10519497	117167	ENSMUSG00000012428	Steap4	STEAP family member 4
10538150	66058	ENSMUSG00000023367	Tmem176a	transmembrane protein 176A
10538503	232016	ENSMUSG00000037973	Itprid1	ITPR interacting domain containing 1
10539449	110935	ENSMUSG00000006269	Atp6v1b1	ATPase, H+ transporting, lysosomal V1 subunit B1
10396849	19363	ENSMUSG00000059060	Rad51b	RAD51 paralog B
10400304	112407	ENSMUSG00000035105	Egln3	egl-9 family hypoxia-inducible factor 3
10401527	16997	ENSMUSG00000002020	Ltbp2	latent transforming growth factor beta binding protein 2
10534152	212996	ENSMUSG00000034040	Galnt17	polypeptide N-acetylgalactosaminyltransferase 17
10400405	18035	ENSMUSG00000021025	Nfkbia	nuclear factor of kappa light polypeptide gene enhancer in B cells inhibitor, alpha
10490903	71934	ENSMUSG00000027555	Car13	carbonic anhydrase 13
10577560	16150	ENSMUSG00000031537	Ikbkb	inhibitor of kappaB kinase beta
10441902	64074	ENSMUSG00000023886	Smoc2	SPARC related modular calcium binding 2
10490923	12349	ENSMUSG00000027562	Car2	carbonic anhydrase 2
10371607	71712	ENSMUSG00000020057	Dram1	DNA-damage regulated autophagy modulator 1
10567335	56209	ENSMUSG00000033917	Gde1	glycerophosphodiester phosphodiesterase 1
10469786	215257	ENSMUSG00000044103	Il36g	interleukin 36G
10490989	12870	ENSMUSG00000003617	Cp	ceruloplasmin
10502552	12722	ENSMUSG00000056025	Clca3a1	chloride channel accessory 3A1
10587799	18828	ENSMUSG00000032372	Plscr2	phospholipid scramblase 2
10582303	13057	ENSMUSG00000006519	Cyba	cytochrome b-245, alpha polypeptide
10429520	17068	ENSMUSG00000034634	Ly6d	lymphocyte antigen 6 complex, locus D
10472289	21353	ENSMUSG00000064289	Tank	TRAF family member-associated Nf-kappa B activator
10385083	216643	ENSMUSG00000020159	Gabrp	gamma-aminobutyric acid (GABA) A receptor, pi
10531972	76074	ENSMUSG00000034438	Gbp8	guanylate-binding protein 8

10580807	16582	ENSMUSG00000031788	Kifc3	kinesin family member C3
10379153	11676	ENSMUSG00000017390	Aldoc	aldolase C, fructose-bisphosphate
10591754	235048	ENSMUSG00000062794	Zfp599	zinc finger protein 599
10445412	18037	ENSMUSG00000023947	Nfkbie	nuclear factor of kappa light polypeptide gene enhancer in B cells inhibitor, epsilon
10384233	319939	ENSMUSG00000020422	Tns3	tensin 3
10544596	65963	ENSMUSG00000029810	Tmem176b	transmembrane protein 176B
10567366	67133	ENSMUSG00000030954	Gp2	glycoprotein 2 (zymogen granule membrane)
10439138	545156	ENSMUSG00000061751	Kalrn	kalirin, RhoGEF kinase
10588037	19659	ENSMUSG00000046402	Rbp1	retinol binding protein 1, cellular
10416689	380924	ENSMUSG00000022026	Olfm4	olfactomedin 4
10439087	224116	ENSMUSG00000035638	Muc20	mucin 20
10442625	16005	ENSMUSG00000046070	Igfals	insulin-like growth factor binding protein, acid labile subunit
10491477	20674	ENSMUSG00000074637	Sox2	SRY (sex determining region Y)-box 2
10493831	20201	ENSMUSG00000056054	S100a8	S100 calcium binding protein A8 (calgranulin A)
10444752	16994	ENSMUSG00000024399	Ltb	lymphotoxin B
10574220	20312	ENSMUSG00000031778	Cx3cl1	chemokine (C-X3-C motif) ligand 1
10436958	209195	ENSMUSG00000022949	Clic6	chloride intracellular channel 6
10499861	20202	ENSMUSG00000056071	S100a9	S100 calcium binding protein A9 (calgranulin B)
10509838	18600	ENSMUSG00000028927	Padi2	peptidyl arginine deiminase, type II
10538187	93695	ENSMUSG00000029816	Gpnmb	glycoprotein (transmembrane) nmb
10563377	54200	ENSMUSG00000003271	Sult2b1	sulfotransferase family, cytosolic, 2B, member 1
10421863	18530	ENSMUSG00000036422	Pcdh8	protocadherin 8
10357155	16324	ENSMUSG00000037035	Inhbb	inhibin beta-B
10417561	268709	ENSMUSG00000021750	Fam107a	family with sequence similarity 107, member A
10457628	71367	ENSMUSG00000047161	Chst9	carbohydrate (N-acetylgalactosamine 4-0) sulfotransferase 9
10469066	74186	ENSMUSG00000026676	Ccdc3	coiled-coil domain containing 3
10547664	56619	ENSMUSG00000030142	Clec4e	C-type lectin domain family 4, member e
10396952	69480	ENSMUSG00000042734	Ttc9	tetratricopeptide repeat domain 9
10355967	252903	ENSMUSG00000054702	Ap1s3	adaptor-related protein complex AP-1, sigma 3
10425053	17972	ENSMUSG00000071715	Ncf4	neutrophil cytosolic factor 4
10348858	51800	ENSMUSG00000026278	Bok	BCL2-related ovarian killer
10541114	70727	ENSMUSG00000030134	Rasgef1a	RasGEF domain family, member 1A
10437073	170765	ENSMUSG00000022941	Ripply3	ripply transcriptional repressor 3

10518300	21938	ENSMUSG00000028599	Tnfrsf1b	tumor necrosis factor receptor superfamily, member 1b
10501608	22329	ENSMUSG00000027962	Vcam1	vascular cell adhesion molecule 1
10458382	12475	ENSMUSG00000051439	Cd14	CD14 antigen
10549276	79362	ENSMUSG00000030256	Bhlhe41	basic helix-loop-helix family, member e41
10523145	20309	ENSMUSG00000029375	Cxcl15	chemokine (C-X-C motif) ligand 15
10445746	58217	ENSMUSG00000042265	Trem1	triggering receptor expressed on myeloid cells 1
10493984	99681	ENSMUSG00000052415	Tchh	trichohyalin
10541614	17474	ENSMUSG00000030144	Clec4d	C-type lectin domain family 4, member d
10559185	55925	ENSMUSG00000031098	Syt8	synaptotagmin VIII
10395103	69675	ENSMUSG00000020674	Pxdn	peroxidasin
10411373	15212	ENSMUSG00000021665	Hexb	hexosaminidase B
10544273	23845	ENSMUSG00000029915	Clec5a	C-type lectin domain family 5, member a
10452316	12266	ENSMUSG00000024164	C3	complement component 3
10523120	20311	ENSMUSG00000029371	Cxcl5	chemokine (C-X-C motif) ligand 5
10458283	381148	ENSMUSG00000073600	Prob1	proline rich basic protein 1
10583071	17392	ENSMUSG00000043613	Mmp3	matrix metallopeptidase 3
10511429	12319	ENSMUSG00000041261	Car8	carbonic anhydrase 8
10542164	232413	ENSMUSG00000053063	Clec12a	C-type lectin domain family 12, member a
10484227	228071	ENSMUSG00000042272	Sestd1	SEC14 and spectrin domains 1
10575598	170737	ENSMUSG00000033545	Znrf1	zinc and ring finger 1
10367822	67844	ENSMUSG00000019832	Rab32	RAB32, member RAS oncogene family
10456492	52662	ENSMUSG00000024544	Ldlrad4	low density lipoprotein receptor class A domain containing 4
10533720	80885	ENSMUSG00000045502	Hcar2	hydroxycarboxylic acid receptor 2
10419096	20390	ENSMUSG00000021795	Sftpd	surfactant associated protein D
10512022	214944	ENSMUSG00000073910	Mob3b	MOB kinase activator 3B
10497590	14013	ENSMUSG00000027684	Mecom	MDS1 and EVI1 complex locus
10450069	630499	ENSMUSG00000067203	H2-K2	histocompatibility 2, K region locus 2
10491091	22035	ENSMUSG00000039304	Tnfsf10	tumor necrosis factor (ligand) superfamily, member 10
10559276	16535	ENSMUSG00000009545	Kcnq1	potassium voltage-gated channel, subfamily Q, member 1
10511779	242341	ENSMUSG00000028238	Atp6v0d2	ATPase, H+ transporting, lysosomal V0 subunit D2
10521111	14184	ENSMUSG00000054252	Fgfr3	fibroblast growth factor receptor 3
10517791	18602	ENSMUSG00000025330	Padi4	peptidyl arginine deiminase, type IV
10484201	545428	ENSMUSG00000044033	Ccdc141	coiled-coil domain containing 141

10388254	11484	ENSMUSG00000020774	Aspa	aspartoacylase
10551293	13107	ENSMUSG00000052974	Cyp2f2	cytochrome P450, family 2, subfamily f, polypeptide 2
10534202	17969	ENSMUSG00000015950	Ncf1	neutrophil cytosolic factor 1
10357115	319901	ENSMUSG00000038702	Dsel	dermatan sulfate epimerase-like
10592044	235135	ENSMUSG00000041737	Tmem45b	transmembrane protein 45b
10466127	107350	ENSMUSG00000075010	AW112010	expressed sequence AW112010
10446739	78785	ENSMUSG00000024059	Clip4	CAP-GLY domain containing linker protein family, member 4
10560575	19698	ENSMUSG00000002983	Relb	avian reticuloendotheliosis viral (v-rel) oncogene related B
10367673	213783	ENSMUSG00000040624	Plekhg1	pleckstrin homology domain containing, family G (with RhoGef domain) member 1
10499854	20193	ENSMUSG00000044080	S100a1	S100 calcium binding protein A1
10587315	14860	ENSMUSG00000032348	Gsta4	glutathione S-transferase, alpha 4
10573115	330812	ENSMUSG00000047747	Rnf150	ring finger protein 150
10464030	11551	ENSMUSG00000033717	Adra2a	adrenergic receptor, alpha 2a
10427052	110310	ENSMUSG00000023039	Krt7	keratin 7
10508074	12986	ENSMUSG00000028859	Csf3r	colony stimulating factor 3 receptor (granulocyte)
10408850	18003	ENSMUSG00000021365	Nedd9	neural precursor cell expressed, developmentally down-regulated gene 9
10437151	16516	ENSMUSG00000062609	Kcnj15	potassium inwardly-rectifying channel, subfamily J, member 15
10492136	13175	ENSMUSG00000027797	Dclk1	doublecortin-like kinase 1
10357003	320311	ENSMUSG00000047496	Rnf152	ring finger protein 152
10368144	21929	ENSMUSG00000019850	Tnfaip3	tumor necrosis factor, alpha-induced protein 3
10597743	13051	ENSMUSG00000052336	Cx3cr1	chemokine (C-X3-C motif) receptor 1
10551883	22177	ENSMUSG00000030579	Tyrobp	TYRO protein tyrosine kinase binding protein
10544638	101214	ENSMUSG00000029817	Tra2a	transformer 2 alpha
10476021	19261	ENSMUSG00000037902	Sirpa	signal-regulatory protein alpha
10363082	14728	ENSMUSG00000062593, ENSMUSG00000112148	Lilrb4a	leukocyte immunoglobulin-like receptor, subfamily B, member 4A
10562234	15451	ENSMUSG00000001249	Hpn	hepsin
10417713	218772	ENSMUSG00000017491	Rarb	retinoic acid receptor, beta
10361186	214791	ENSMUSG00000016262	Sertad4	SERTA domain containing 4
10530841	29817	ENSMUSG00000036256	Igfbp7	insulin-like growth factor binding protein 7
10463599	18034	ENSMUSG00000025225	Nfkb2	nuclear factor of kappa light polypeptide gene enhancer in B cells 2, p49/p100
10518147	14726	ENSMUSG00000028583	Pdpn	podoplanin
10347291	12765	ENSMUSG00000026180	Cxcr2	chemokine (C-X-C motif) receptor 2

10444268	21355	ENSMUSG00000024339	Tap2	transporter 2, ATP-binding cassette, sub-family B (MDR/TAP)
10596148	22041	ENSMUSG00000032554	Trf	transferrin
10450501	21926	ENSMUSG00000024401	Tnf	tumor necrosis factor
10407803	83924	ENSMUSG00000021306	Gpr137b	G protein-coupled receptor 137B
10502622	23844	ENSMUSG00000028255	Clca1	chloride channel accessory 1
10429524	78725	ENSMUSG00000101026	Ly6g6g	lymphocyte antigen 6 complex, locus G6G
10450484	11629	ENSMUSG00000024397	Aif1	allograft inflammatory factor 1
10423024	75568	ENSMUSG00000039676	Capsl	calcyphosine-like
10563858	14407	ENSMUSG00000055026	Gabrg3	gamma-aminobutyric acid (GABA) A receptor, subunit gamma 3
10435565	15163	ENSMUSG00000022831	Hcls1	hematopoietic cell specific Lyn substrate 1
10607467	20229	ENSMUSG00000025283	Sat1	spermidine/spermine N1-acetyl transferase 1
10355214	15926	ENSMUSG00000025950	Idh1	isocitrate dehydrogenase 1 (NADP+), soluble
10450145	16912	ENSMUSG00000096727	Psmb9	proteasome (prosome, macropain) subunit, beta type 9 (large multifunctional peptidase 2)
10401238	12192	ENSMUSG00000021127	Zfp36l1	zinc finger protein 36, C3H type-like 1
10351679	12523	ENSMUSG00000038147	Cd84	CD84 antigen
10508772	14191	ENSMUSG00000028874	Fgr	FGR proto-oncogene, Src family tyrosine kinase
10389231	20302	ENSMUSG00000000982	Ccl3	chemokine (C-C motif) ligand 3
10547621	11810	ENSMUSG00000040613	Apobec1	apolipoprotein B mRNA editing enzyme, catalytic polypeptide 1
10464167	11554	ENSMUSG00000035283	Adrb1	adrenergic receptor, beta 1
10487597	16176	ENSMUSG00000027398	Il1b	interleukin 1 beta
10435457	80285	ENSMUSG00000022906	Parp9	poly (ADP-ribose) polymerase family, member 9
10444258	16913	ENSMUSG00000024338	Psmb8	proteasome (prosome, macropain) subunit, beta type 8 (large multifunctional peptidase 7)
10540472	20893	ENSMUSG00000030103	Bhlhe40	basic helix-loop-helix family, member e40
10541605	56620	ENSMUSG00000023349	Clec4n	C-type lectin domain family 4, member n
10458828	12583	ENSMUSG00000033022	Cdo1	cysteine dioxygenase 1, cytosolic
10580635	104158	ENSMUSG00000056973	Ces1d	carboxylesterase 1D
10436100	245195	ENSMUSG00000022651	Retnlg	resistin like gamma
10494271	13040	ENSMUSG00000038642	Ctss	cathepsin S
10602198	18481	ENSMUSG00000031284	Pak3	p21 (RAC1) activated kinase 3
10527638	11690	ENSMUSG00000060063	Alox5ap	arachidonate 5-lipoxygenase activating protein
10349782	74137	ENSMUSG00000009772	Nuak2	NUAK family, SNF1-like kinase, 2

10441003	12394	ENSMUSG00000022952	Runx1	runt related transcription factor 1
10392815	140497	ENSMUSG00000044811	Cd300c2	CD300C molecule 2
10371111	27375	ENSMUSG00000034917	Tjp3	tight junction protein 3
10473024	228061	ENSMUSG00000042410	Agps	alkylglycerone phosphate synthase
10419170	70561	ENSMUSG00000021830	Txndc16	thioredoxin domain containing 16
10462442	77125	ENSMUSG00000024810	Il33	interleukin 33
10379530	20293	ENSMUSG00000035352	Ccl12	chemokine (C-C motif) ligand 12
10505911	242523	ENSMUSG00000043753	Dmrta1	doublesex and mab-3 related transcription factor like family A1
10436608	13052	ENSMUSG00000022865	Cxadr	coxsackie virus and adenovirus receptor
10444814	15015	ENSMUSG00000035929	H2-Q4	histocompatibility 2, Q region locus 4
10569877	69189	ENSMUSG00000013974	Mcemp1	mast cell expressed membrane protein 1
10577641	69068	ENSMUSG00000056313	Tcim	transcriptional and immune response regulator
10498992	24088	ENSMUSG00000027995	Tlr2	toll-like receptor 2
10449741	17691	ENSMUSG00000024042	Sik1	salt inducible kinase 1
10446253	22324	ENSMUSG00000034116	Vav1	vav 1 oncogene
10386033	245827	ENSMUSG00000055333	Fat2	FAT atypical cadherin 2
10387890	66102	ENSMUSG00000018920	Cxcl16	chemokine (C-X-C motif) ligand 16
10384458	56193	ENSMUSG00000020120	Plek	pleckstrin
10356475	320982	ENSMUSG00000049866	Arl4c	ADP-ribosylation factor-like 4C
10376060	16362	ENSMUSG00000018899	Irf1	interferon regulatory factor 1
10430660	18591	ENSMUSG00000000489	Pdgfb	platelet derived growth factor, B polypeptide
10586491	13143	ENSMUSG00000032380	Dapk2	death-associated protein kinase 2
10497372	381484	ENSMUSG00000078780	Gm5150	predicted gene 5150
10421697	210808	ENSMUSG00000044350	Lacc1	laccase domain containing 1
10599174	16164	ENSMUSG00000017057	Il13ra1	interleukin 13 receptor, alpha 1
10385583	17001	ENSMUSG00000020377	Ltc4s	leukotriene C4 synthase
10456756	207259	ENSMUSG00000044646	Zbtb7c	zinc finger and BTB domain containing 7C
10583008	12364	ENSMUSG00000025887	Casp12	caspase 12
10557326	16190	ENSMUSG00000030748	Il4ra	interleukin 4 receptor, alpha
10397645	14744	ENSMUSG00000021886	Gpr65	G-protein coupled receptor 65
10478698	14049	ENSMUSG00000017897	Eya2	EYA transcriptional coactivator and phosphatase 2
10389222	20305	ENSMUSG00000018927	Ccl6	chemokine (C-C motif) ligand 6
10556616	74424	ENSMUSG00000030650	Tmc5	transmembrane channel-like gene family 5

10466659	14544	ENSMUSG00000058624	Gda	guanine deaminase
10523156	20310	ENSMUSG00000058427	Cxcl2	chemokine (C-X-C motif) ligand 2
10542470	56615	ENSMUSG00000008540	Mgst1	microsomal glutathione S-transferase 1
10409314	17702	ENSMUSG00000021469	Msx2	msh homeobox 2
10360070	14127	ENSMUSG00000058715	Fcer1g	Fc receptor, IgE, high affinity I, gamma polypeptide
10589654	235633	ENSMUSG00000044037	Als2cl	ALS2 C-terminal like
10477250	15162	ENSMUSG00000003283	Hck	hemopoietic cell kinase
10517517	12259	ENSMUSG00000036887	C1qa	complement component 1, q subcomponent, alpha polypeptide
10478525	67701	ENSMUSG00000017723	Wfdc2	WAP four-disulfide core domain 2
10536505	17295	ENSMUSG00000009376	Met	met proto-oncogene
10521892	20531	ENSMUSG00000029188	Slc34a2	solute carrier family 34 (sodium phosphate), member 2
10387536	12514	ENSMUSG00000018774	Cd68	CD68 antigen
10582985	12362	ENSMUSG00000025888	Casp1	caspase 1
10550509	21946	ENSMUSG00000030413	Pglyrp1	peptidoglycan recognition protein 1
10492586	71619	ENSMUSG00000098207	Arl14	ADP-ribosylation factor-like 14
10598004	12768	ENSMUSG00000025804	Ccr1	chemokine (C-C motif) receptor 1
10517513	12262	ENSMUSG00000036896	C1qc	complement component 1, q subcomponent, C chain
10392808	217305	ENSMUSG00000034641	Cd300ld	CD300 molecule like family member d
10575095	15118	ENSMUSG00000031910	Has3	hyaluronan synthase 3
10572679	234407	ENSMUSG00000034807	Colgalt1	collagen beta(1-O)galactosyltransferase 1
10461614	73656	ENSMUSG00000079419	Ms4a6c	membrane-spanning 4-domains, subfamily A, member 6C
10461721	17476	ENSMUSG00000046805	Mpeg1	macrophage expressed gene 1
10583203	18689	ENSMUSG00000031802	Phxr4	per-hexamer repeat gene 4
10444821	15016	ENSMUSG00000055413	H2-Q5	histocompatibility 2, Q region locus 5
10483000	16420	ENSMUSG00000026971	Itgb6	integrin beta 6
10418506	192187	ENSMUSG00000042286	Stab1	stabilin 1
10582997	12363	ENSMUSG00000033538	Casp4	caspase 4, apoptosis-related cysteine peptidase
10530615	433904	ENSMUSG00000029153	Ociad2	OCIA domain containing 2
10363070	14727	ENSMUSG00000112023	Lilrb4b	leukocyte immunoglobulin-like receptor, subfamily B, member 4B
10501164	12977	ENSMUSG00000014599	Csf1	colony stimulating factor 1 (macrophage)
10548892	11857	ENSMUSG00000030220	Arhgdib	Rho, GDP dissociation inhibitor (GDI) beta
10490159	65112	ENSMUSG00000038400	Pmepa1	prostate transmembrane protein, androgen induced 1
10592084	20443	ENSMUSG00000032038	St3gal4	ST3 beta-galactoside alpha-2,3-sialyltransferase 4

10392845	246746	ENSMUSG00000047798	Cd300lf	CD300 molecule like family member F
10478633	17395	ENSMUSG00000017737	Mmp9	matrix metallopeptidase 9
10356764	71874	ENSMUSG00000034159	Mab21l4	mab-21-like 4
10581378	19171	ENSMUSG00000031897	Psmb10	proteasome (prosome, macropain) subunit, beta type 10
10602081	78789	ENSMUSG00000031430	Vsig1	V-set and immunoglobulin domain containing 1
10417544	93732	ENSMUSG00000021751	Acox2	acyl-Coenzyme A oxidase 2, branched chain
10462881	107221	ENSMUSG00000054200	Ffar4	free fatty acid receptor 4
10358434	18783	ENSMUSG00000056220	Pla2g4a	phospholipase A2, group IVA (cytosolic, calcium-dependent)
10590631	12772	ENSMUSG00000049103	Ccr2	chemokine (C-C motif) receptor 2
10598013	12774	ENSMUSG00000079227	Ccr5	chemokine (C-C motif) receptor 5
10501048	69206	ENSMUSG00000091575	2010016I18Rik	RIKEN cDNA 2010016I18 gene
10347335	18173	ENSMUSG00000026177	Slc11a1	solute carrier family 11 (proton-coupled divalent metal ion transporters), member 1
10500335	14129	ENSMUSG00000015947	Fcgr1	Fc receptor, IgG, high affinity I
10458314	72512	ENSMUSG00000024349	Sting1	stimulator of interferon response cGAMP interactor 1
10351224	14067	ENSMUSG00000026579	F5	coagulation factor V
10383502	80879	ENSMUSG00000025161	Slc16a3	solute carrier family 16 (monocarboxylic acid transporters), member 3
10450325	14962	ENSMUSG00000090231	Cfb	complement factor B
10500656	630146	ENSMUSG00000086564	Cd101	CD101 antigen
10467153	240638	ENSMUSG00000009378	Slc16a12	solute carrier family 16 (monocarboxylic acid transporters), member 12
10381096	16010	ENSMUSG00000017493	Igfbp4	insulin-like growth factor binding protein 4
10601385	279572	ENSMUSG00000033777	Tlr13	toll-like receptor 13
10501063	12508	ENSMUSG00000040747	Cd53	CD53 antigen
10443980	17916	ENSMUSG00000024300	Myo1f	myosin IF
10434559	13845	ENSMUSG00000005958	Ephb3	Eph receptor B3
10412267	16398	ENSMUSG00000015533	Itga2	integrin alpha 2
10519140	26561	ENSMUSG00000029061	Mmp23	matrix metallopeptidase 23
10559486	52855	ENSMUSG00000055541	Lair1	leukocyte-associated Ig-like receptor 1
10349295	81879	ENSMUSG00000026380	Tfcp2l1	transcription factor CP2-like 1
10420891	219151	ENSMUSG00000034463	Scara3	scavenger receptor class A, member 3
10514466	16476	ENSMUSG00000052684	Jun	jun proto-oncogene
10412562	286940	ENSMUSG00000025278	Flnb	filamin, beta
10443730	11307	ENSMUSG00000024030	Abcg1	ATP binding cassette subfamily G member 1
10499189	80891	ENSMUSG00000015852	Fcrls	Fc receptor-like S, scavenger receptor

10579636	72054	ENSMUSG00000003484	Cyp4f18	cytochrome P450, family 4, subfamily f, polypeptide 18
10456357	58801	ENSMUSG00000024521	Pmaip1	phorbol-12-myristate-13-acetate-induced protein 1
10466521	14537	ENSMUSG00000038843	Gcnt1	glucosaminyl (N-acetyl) transferase 1, core 2
10456005	16149	ENSMUSG00000024610	Cd74	CD74 antigen (invariant polypeptide of major histocompatibility complex, class II antigen-associated)
10442565	71893	ENSMUSG00000019320	Noxo1	NADPH oxidase organizer 1
10458303	68545	ENSMUSG00000073599	Ecscr	endothelial cell surface expressed chemotaxis and apoptosis regulator
10538791	414084	ENSMUSG00000044162	Tnip3	TNFAIP3 interacting protein 3
10369615	19073	ENSMUSG00000020077	Srgn	serglycin
10450154	14960	ENSMUSG00000036594	H2-Aa	histocompatibility 2, class II antigen A, alpha
10583669	382062	ENSMUSG00000057191	AB124611	cDNA sequence AB124611
10425808	12257	ENSMUSG00000041736	Tspo	translocator protein
10604961	14396	ENSMUSG00000031343	Gabra3	gamma-aminobutyric acid (GABA) A receptor, subunit alpha 3
10357472	12767	ENSMUSG00000045382	Cxcr4	chemokine (C-X-C motif) receptor 4
10446282	13733	ENSMUSG00000004730	Adgre1	adhesion G protein-coupled receptor E1
10360806	12334	ENSMUSG00000026509	Capn2	calpain 2
10475653	26458	ENSMUSG00000027359	Slc27a2	solute carrier family 27 (fatty acid transporter), member 2
10568024	12721	ENSMUSG00000030707	Coro1a	coronin, actin binding protein 1A
10445293	27226	ENSMUSG00000023913	Pla2g7	phospholipase A2, group VII (platelet-activating factor acetylhydrolase, plasma)
10405566	328258	ENSMUSG00000021509	Slc25a48	solute carrier family 25, member 48
10562192	18301	ENSMUSG00000009687	Fxyd5	FXYD domain-containing ion transport regulator 5
10363445	74048	ENSMUSG00000020101	Vsir	V-set immunoregulatory receptor
10548375	56644	ENSMUSG00000079293	Clec7a	C-type lectin domain family 7, member a
10415052	17387	ENSMUSG00000000957	Mmp14	matrix metallopeptidase 14 (membrane-inserted)
10445112	24108	ENSMUSG00000035186	Ubd	ubiquitin D
10562709	12489	ENSMUSG00000004609	Cd33	CD33 antigen
10487321	66552	ENSMUSG00000027366	Sppl2a	signal peptide peptidase like 2A
10565775	67800	ENSMUSG00000030747	Dgat2	diacylglycerol O-acyltransferase 2
10404606	17084	ENSMUSG00000021423	Ly86	lymphocyte antigen 86
10391066	16667	ENSMUSG00000035557	Krt17	keratin 17
10512470	12517	ENSMUSG00000028459	Cd72	CD72 antigen
10548817	66857	ENSMUSG00000030214	Plbd1	phospholipase B domain containing 1
10465861	16319	ENSMUSG00000024660	Incenp	inner centromere protein

10541885	20276	ENSMUSG00000030340	Scnn1a	sodium channel, nonvoltage-gated 1 alpha
10592050	12023	ENSMUSG00000032033	Barx2	BarH-like homeobox 2
10364262	16414	ENSMUSG00000000290	Itgb2	integrin beta 2
10542050	381812	ENSMUSG00000061414	Cracr2a	calcium release activated channel regulator 2A
10471844	59126	ENSMUSG00000026749	Nek6	NIMA (never in mitosis gene a)-related expressed kinase 6
10404250	195208	ENSMUSG00000035910	Dcdc2a	doublecortin domain containing 2a
10427336	105855	ENSMUSG00000022488	Nckap1l	NCK associated protein 1 like
10461558	65221	ENSMUSG00000024737	Slc15a3	solute carrier family 15, member 3
10493267	16800	ENSMUSG00000028059	Arhgef2	rho/rac guanine nucleotide exchange factor (GEF) 2
10597518	21813	ENSMUSG00000032440	Tgfbr2	transforming growth factor, beta receptor II
10358027	13710	ENSMUSG00000003051	Elf3	E74-like factor 3
10424686	223631	ENSMUSG00000047728	Ly6g2	lymphocyte antigen 6 complex, locus G2
10566709	244202	ENSMUSG00000049709	Nlrp10	NLR family, pyrin domain containing 10
10413047	18792	ENSMUSG00000021822	Plau	plasminogen activator, urokinase
10439268	209200	ENSMUSG00000049502	Dtx3l	deltex 3-like, E3 ubiquitin ligase
10349968	12654	ENSMUSG00000064246	Chil1	chitinase-like 1
10560685	12051	ENSMUSG00000053175	Bcl3	B cell leukemia/lymphoma 3
10464529	27060	ENSMUSG00000001750	Tcirg1	T cell, immune regulator 1, ATPase, H+ transporting, lysosomal V0 protein A3
10560624	11816	ENSMUSG00000002985	Apoe	apolipoprotein E
10473809	20375	ENSMUSG00000002111	Spi1	spleen focus forming virus (SFFV) proviral integration oncogene
10557862	16409	ENSMUSG00000030786	Itgam	integrin alpha M
10414360	16854	ENSMUSG00000050335	Lgals3	lectin, galactose binding, soluble 3
10382912	53860	ENSMUSG00000059248	Septin9	septin 9
10557895	16411	ENSMUSG00000030789	Itgax	integrin alpha X
10412773	218756	ENSMUSG00000021733	Slc4a7	solute carrier family 4, sodium bicarbonate cotransporter, member 7
10461622	69774	ENSMUSG00000024677	Ms4a6b	membrane-spanning 4-domains, subfamily A, member 6B
10358224	19264	ENSMUSG00000026395	Ptprc	protein tyrosine phosphatase, receptor type, C
10494761	242122	ENSMUSG00000051076	Vtcn1	V-set domain containing T cell activation inhibitor 1
10576911	13642	ENSMUSG00000001300	Efnb2	ephrin B2
10413928	69069	ENSMUSG00000041707	Tmem273	transmembrane protein 273
10351509	246256	ENSMUSG00000059089	Fcgr4	Fc receptor, IgG, low affinity IV
10359034	104009	ENSMUSG00000033684	Qsox1	quiescin Q6 sulfhydryl oxidase 1
10583056	17381	ENSMUSG00000049723	Mmp12	matrix metallopeptidase 12

10539739	67855	ENSMUSG00000033508	Asprv1	aspartic peptidase, retroviral-like 1
10437210	56175	ENSMUSG00000040605	Bace2	beta-site APP-cleaving enzyme 2
10519951	212167	ENSMUSG00000039934	Gsap	gamma-secretase activating protein
10404422	20708	ENSMUSG00000042842	Serpinb6b	serine (or cysteine) peptidase inhibitor, clade B, member 6b
10526783	381680	ENSMUSG00000047592	Nxpe5	neurexophilin and PC-esterase domain family, member 5
10352756	226856	ENSMUSG00000026623	Lpgat1	lysophosphatidylglycerol acyltransferase 1
10571467	68797	ENSMUSG00000031595	Pdgfrl	platelet-derived growth factor receptor-like
10466200	109225	ENSMUSG00000024672	Ms4a7	membrane-spanning 4-domains, subfamily A, member 7
10545168	56753	ENSMUSG00000051397	Tacstd2	tumor-associated calcium signal transducer 2
10474437	68201	ENSMUSG00000027160	Ccdc34	coiled-coil domain containing 34
10365991	13516	ENSMUSG00000019936	Epyc	epiphycan
10402268	19141	ENSMUSG00000021190	Lgmn	legumain
10430006	72027	ENSMUSG00000063354	Slc39a4	solute carrier family 39 (zinc transporter), member 4
10545569	19695	ENSMUSG00000030017	Reg3g	regenerating islet-derived 3 gamma
10517165	23833	ENSMUSG00000000682	Cd52	CD52 antigen
10547657	12267	ENSMUSG00000040552	C3ar1	complement component 3a receptor 1
10472538	241452	ENSMUSG00000027068	Dhrs9	dehydrogenase/reductase (SDR family) member 9
10435930	271375	ENSMUSG00000090176	Cd200r2	Cd200 receptor 2
10561047	232983	ENSMUSG00000060188	Cxcl17	chemokine (C-X-C motif) ligand 17
10457640	20195	ENSMUSG00000027907	S100a11	S100 calcium binding protein A11
10466210	68774	ENSMUSG00000024679	Ms4a6d	membrane-spanning 4-domains, subfamily A, member 6D
10375065	268396	ENSMUSG00000040711	Sh3pxd2b	SH3 and PX domains 2B
10529824	19126	ENSMUSG00000029086	Prom1	prominin 1
10428955	331063	ENSMUSG00000056293	Gsdmc2	gasdermin C2
10476545	228677	ENSMUSG00000039092	Sptlc3	serine palmitoyltransferase, long chain base subunit 3
10425092	72318	ENSMUSG00000018008	Cyth4	cytohesin 4
10401109	14776	ENSMUSG00000042808	Gpx2	glutathione peroxidase 2
10429029	11514	ENSMUSG00000022376	Adcy8	adenylate cyclase 8
10519324	12571	ENSMUSG00000040274	Cdk6	cyclin-dependent kinase 6
10464251	226255	ENSMUSG00000054843	Atrnl1	attractin like 1
10517508	12260	ENSMUSG00000036905	C1qb	complement component 1, q subcomponent, beta polypeptide
10375145	16822	ENSMUSG00000002699	Lcp2	lymphocyte cytosolic protein 2
10508663	16792	ENSMUSG00000028581	Laptm5	lysosomal-associated protein transmembrane 5

10510129	20148	ENSMUSG00000066026	Dhrs3	dehydrogenase/reductase (SDR family) member 3
10389143	276950	ENSMUSG00000035208	Slfn8	schlafen 8
10425161	16852	ENSMUSG00000068220	Lgals1	lectin, galactose binding, soluble 1
10496324	67547	ENSMUSG00000053897	Slc39a8	solute carrier family 39 (metal ion transporter), member 8
10488973	74562	ENSMUSG00000013338	Fer1l4	fer-1-like 4 (C. elegans)
10414065	11752	ENSMUSG00000021950	Anxa8	annexin A8
10417212	223272	ENSMUSG00000032925	Itgbl1	integrin, beta-like 1
10412100	26401	ENSMUSG00000021754	Map3k1	mitogen-activated protein kinase kinase kinase 1
10356271	381287	ENSMUSG00000089844	A530032D15Rik	RIKEN cDNA A530032D15Rik gene
10379636	20558	ENSMUSG00000000204	Slfn4	schlafen 4
10545101	54486	ENSMUSG00000029919	Hpgds	hematopoietic prostaglandin D synthase
10372648	17105	ENSMUSG00000069516	Lyz2	lysozyme 2
10398665	21928	ENSMUSG00000021281	Tnfaip2	tumor necrosis factor, alpha-induced protein 2
10398907	104759	ENSMUSG00000052160	Pld4	phospholipase D family, member 4
10508465	17357	ENSMUSG00000047945	Marcksl1	MARCKS-like 1
10404913	67252	ENSMUSG00000021373	Cap2	CAP, adenylate cyclase-associated protein, 2 (yeast)
10502240	114249	ENSMUSG00000040998	Npnt	nephronectin
10574259	14766	ENSMUSG00000031785	Adgrg1	adhesion G protein-coupled receptor G1
10582162	72042	ENSMUSG00000031827	Cotl1	coactosin-like 1 (Dictyostelium)
10444291	14961	ENSMUSG00000073421	H2-Ab1	histocompatibility 2, class II antigen A, beta 1
10512999	381524	ENSMUSG00000086712	Mexis	macrophage expressed LXRa(NR1H3)-dependent amplifier of Abca1 transcription lncRNA
10435937	74603	ENSMUSG00000036172	Cd200r3	CD200 receptor 3
10389214	20308	ENSMUSG00000019122	Ccl9	chemokine (C-C motif) ligand 9
10378216	53313	ENSMUSG00000020788	Atp2a3	ATPase, Ca++ transporting, ubiquitous
10439249	547253	ENSMUSG00000034422	Parp14	poly (ADP-ribose) polymerase family, member 14
10495675	14066	ENSMUSG00000028128	F3	coagulation factor III
10379633	20555	ENSMUSG00000078763	Slfn1	schlafen 1
10429528	76486	ENSMUSG00000044678	Ly6k	lymphocyte antigen 6 complex, locus K
10570957	20377	ENSMUSG00000031548	Sfrp1	secreted frizzled-related protein 1
10564818	16790	ENSMUSG00000039062	Anpep	alanyl (membrane) aminopeptidase
10534862	18542	ENSMUSG00000029718	Pcolce	procollagen C-endopeptidase enhancer protein

10561187	12587	ENSMUSG00000089661	Mia	MIA SH3 domain containing
10366446	216350	ENSMUSG00000034127	Tspan8	tetraspanin 8
10570855	18791	ENSMUSG00000031538	Plat	plasminogen activator, tissue
10574149	434341	ENSMUSG00000074151	Nlrc5	NLR family, CARD domain containing 5
10450344	12263	ENSMUSG00000024371	C2	complement component 2 (within H-2S)
10467258	226101	ENSMUSG00000048612	Myof	myoferlin
10541564	73149	ENSMUSG00000043832	Clec4a3	C-type lectin domain family 4, member a3
10441244	52793	ENSMUSG00000022938	Fam3b	FAM3 metabolism regulating signaling molecule B
10544660	71720	ENSMUSG00000029822	Osbpl3	oxysterol binding protein-like 3
10456071	12978	ENSMUSG00000024621	Csf1r	colony stimulating factor 1 receptor
10450374	110956	NA	D17H6S56E-5	DNA segment, Chr 17, human D6S56E 5
10514347	12579	ENSMUSG00000073802	Cdkn2b	cyclin dependent kinase inhibitor 2B
10398075	20716	ENSMUSG00000021091	Serpina3n	serine (or cysteine) peptidase inhibitor, clade A, member 3N
10571312	319520	ENSMUSG00000031530	Dusp4	dual specificity phosphatase 4
10595211	12816	ENSMUSG00000032332	Col12a1	collagen, type XII, alpha 1
10538253	56524	ENSMUSG00000038388	Pals2	protein associated with LIN7 2, MAGUK family member
10567580	80719	ENSMUSG00000035004	Igsf6	immunoglobulin superfamily, member 6
10528332	242864	ENSMUSG00000044968	Napepld	N-acyl phosphatidylethanolamine phospholipase D
10542911	209086	ENSMUSG00000047735	Samd9l	sterile alpha motif domain containing 9-like
10403834	20379	ENSMUSG00000021319	Sfrp4	secreted frizzled-related protein 4
10558961	64540	ENSMUSG00000025511	Tspan4	tetraspanin 4
10548879	17313	ENSMUSG00000030218	Mgp	matrix Gla protein
10391052	16664	ENSMUSG00000045545	Krt14	keratin 14
10380862	19049	ENSMUSG00000061718	Ppp1r1b	protein phosphatase 1, regulatory inhibitor subunit 1B
10416837	16365	ENSMUSG00000022126	Acod1	aconitate decarboxylase 1
10443494	74116	ENSMUSG00000024011	Pi16	peptidase inhibitor 16
10444244	21354	ENSMUSG00000037321	Tap1	transporter 1, ATP-binding cassette, sub-family B (MDR/TAP)
10420488	219132	ENSMUSG00000068245	Phf11d	PHD finger protein 11D
10472235	76747	ENSMUSG00000026989	Dapl1	death associated protein-like 1
10351658	12506	ENSMUSG00000015355	Cd48	CD48 antigen
10481627	16819	ENSMUSG00000026822	Lcn2	lipocalin 2
10357660	213006	ENSMUSG00000059149	Mfsd4a	major facilitator superfamily domain containing 4A
